# Genome-wide identification of *GhAOX* gene family and its expression and regulation in cotton CMS line Jin A

**DOI:** 10.3389/fpls.2026.1898123

**Published:** 2026-08-14

**Authors:** Jinlong Zhang, Li Zhang, Yuekai Su, Jinjiang Shi, Junfang Yang, Xiuqing Yang, Jinling Huang, Yunfang Qu

**Affiliations:** 1 College of Agriculture, Shanxi Agricultural University, Jinzhong, China; 2 College of Forestry, Shanxi Agricultural University, Jinzhong, China

**Keywords:** alternative oxidase, AOX isoforms, bioinformatics analysis, cytoplasmic male sterile, protein post-translational modification

## Abstract

**Introduction:**

Alternative oxidase (AOX) is a core functional protein that mitigates excessive mitochondrial reactive oxygen species (ROS) accumulation and modulates plant stress adaptation, and its biological activity is tightly controlled by sophisticated regulatory networks.The cotton cytoplasmic male sterile (CMS) line Jin A represents an optimal model for dissecting the functional characteristics of *GhAOX* genes, as it exhibits pronounced ROS overaccumulation and aberrant *GhAOX* transcriptional profiles in anthers during the critical developmental stage of pollen mother cell degeneration, relative to its maintainer Jin B.

**Methods:**

We conducted a genome-wide identification of the *GhAOX* gene family in upland cotton (*Gossypium hirsutum*) and systematically characterized the molecular features of *GhAOX* members in Jin A at the transcriptional, translational, post-translational modification, and gene structural levels.

**Results:**

A total of eight *GhAOX* genes were identified, among which *GhAOX2b1/2* and *GhAOX2c1/2* displayed anther-preferential expression patterns and were significantly upregulated in response to multiple abiotic stresses, including osmotic stress (PEG), salinity (NaCl), cold (4℃) and heat (37℃). Functional validation via overexpression and virus-induced gene silencing (VIGS) assays verified the ROS-scavenging capacity of *GhAOX*. Notably, in Jin A, despite elevated *GhAOX* protein abundance, its enzymatic activity was substantially compromised, which was attributed to perturbed transcriptional regulation, isoform-specific differential activation by tricarboxylic acid (TCA) cycle intermediates, and reduced abundance of mitochondrial effector molecules.

**Discussion:**

This dysregulation was likely implicated in the aberrant ROS accumulation underlying the pollen abortion phenotype in Jin A. Collectively, this study establishes a comprehensive molecular framework for elucidating the multi-level regulatory mechanisms of AOX in response to intracellular microenvironmental perturbations and uncovers its pivotal role in regulating reproductive fitness in upland cotton.

## 1 Introduction

Higher plant mitochondria harbors two functionally distinct electron transport pathways that mediate electron transfer from ubiquinone to molecular oxygen: the canonical cytochrome c oxidase (COX) pathway and the alternative oxidase (AOX) pathway (Grabelnych et al., 2004; Selinski et al., 2018a). The COX pathway consists of evolutionarily conserved mitochondrial Complexes I-IV, which are also present in metazoans, and tightly couples electron flux to transmembrane proton translocation, thereby driving ATP synthesis. In contrast, the cyanide-resistant AOX pathway uncouples electron transport from ATP production by bypassing two proton-translocating sites within the mitochondrial respiratory chain. This process dissipates excess free energy as heat and maintains sustained electron flow when the canonical COX pathway is functionally restricted (Moore and Siedow, 1991; Gupta et al., 2012). Although the energy-dissipating nature of the AOX pathway appears metabolically uneconomical, it fulfills essential physiological roles in thermogenic plant species, such as members of the Araceae and Nelumbonaceae. AOX-mediated thermogenesis facilitates floral development and ensures successful pollination under low-temperature conditions (Wagner et al., 2008; Barthlott et al., 2009; Zheng et al., 2022; Tanimoto et al., 2024). Dynamic electron partitioning between the COX and AOX pathways, along with their synergistic regulatory crosstalk, is critical for balancing cellular energy metabolism and maintaining mitochondrial redox homeostasis in plants under variable environmental conditions (Sunil and Raghavendra, 2017).

AOX is a homodimeric di-iron carboxylate protein anchored in the mitochondrial inner membrane, which is encoded by a small, evolutionarily divergent nuclear gene family (Affourtit et al., 2002; Selinski et al., 2018a; Yang et al., 2019). In plants, the AOX family generally contains 1–6 paralogous genes with highly variable duplication and retention patterns across species (Costa et al., 2014; Zhang et al., 2024). Plastoquinol oxidase (AOX4/PTOX), a chloroplast-targeted structural homolog, represents the closest phylogenetic counterpart of mitochondrial AOX. Based on the canonical classification criteria, plant AOXs are divided into two major subfamilies, *AOX1* and *AOX2* (Costa et al., 2014). Dicotyledons typically retain both subfamilies, whereas most monocot species (excluding basal lineages) harbor only the *AOX1* subfamily (Frederico et al., 2009; Costa et al., 2017). Despite evolutionary divergence, core functional domains and critical amino acid residues are highly conserved among all AOX isoforms, as validated by structural and functional characterizations of AOX proteins from *Trypanosoma brucei* and *Arabidopsis thaliana* (Andersson and Nordlund, 1999; Berthold et al., 2000; Shiba et al., 2019; Young et al., 2020; Xu et al., 2021; Yamasaki et al., 2021; Sankar et al., 2022).

The primary physiological function of AOX is to catalyze molecular oxygen reduction, thereby alleviating mitochondrial reactive oxygen species (ROS) overaccumulation (Moore et al., 2002; Yokoyama, 2023; Zhu et al., 2025). Both mitochondrial AOX and chloroplastic PTOX facilitate electron partitioning toward O_2_, preventing excessive ROS generation triggered by impaired oxidative phosphorylation and photophosphorylation, respectively (Mittler, 2002; Cvetkovska and Vanlerberghe, 2012; Dahal and Vanlerberghe, 2017). Under adverse environmental conditions, the transcription of *AOX* genes, especially *AOX1* subfamily members, is markedly induced to restrict mitochondrial ROS burst (Murik et al., 2019; He et al., 2019, 2021; Fedotova et al., 2023; Manbir et al., 2022; Aziz et al., 2022; Qiao et al., 2023; Analin et al., 2024). Genetic evidence further supports this antioxidant function: *Arabidopsis AtAOX1a* knockout mutants exhibit enhanced stress sensitivity, while *AtAOX1a*-overexpressing lines accumulate fewer ROS under stress conditions (Giraud et al., 2008; Smith et al., 2009; Sweetman et al., 2019). Furthermore, AOX acts as a core functional component and biomarker of mitochondrial retrograde regulation (MRR), a vital signaling pathway that transmits mitochondrial functional status to the nucleus (Butow and Avadhani, 2004; Clifton et al., 2005; Rhoads and Subbaiah, 2007). Robust transcriptional activation of *AOX* genes is a hallmark MRR response. In particular, *AtAOX1a* serves as a canonical MRR reporter in *Arabidopsis*, which is strongly induced by mitochondrial dysfunction to sustain electron flux and minimize ROS production in defective respiratory chains (Clifton et al., 2005).

*in vivo*, AOX activity is not strictly correlated with protein abundance, particularly under high-light stress conditions (Florez-Sarasa et al., 2016). The regulation of AOX is governed by a multilayered regulatory network, encompassing transcriptional control, post-translational modifications (including redox status and sensitivity to 2-oxo acids), and the abundance of key mitochondrial metabolites (Selinski et al., 2017, 2018a). The majority of transcription factors identified to date as regulators of *AOX* expression, including ABI4, MYB29, and auxin signaling components, function as transcriptional repressors (Giraud et al., 2009; Ng et al., 2013a; Van Aken et al., 2013; Zhang et al., 2017). Consequently, *AtAOX1a* expression is strongly suppressed under non-stressed, basal physiological conditions (Ho et al., 2008). Moreover, evolutionary divergence in the regulatory mechanisms between the *AOX1* and *AOX2* subfamilies is postulated to underpin their distinct stress-responsive expression patterns (Pu et al., 2015).

Beyond transcriptional control, AOX activity is predominantly modulated via post-translational mechanisms relying on three conserved N-terminal cysteine residues (CysI, CysII, and CysIII) (Millar et al., 1993; Selinski et al., 2016, 2017, 2018a). Two synergistic molecular mechanisms govern AOX activation status. First, redox-dependent modulation: under oxidizing conditions, intermolecular disulfide bond formation between CysI residues of two AOX monomers generates catalytically inactive oxidized dimers. Under reducing conditions, this disulfide bond is reduced to form non-covalent, enzymatically active AOX dimers. The thioredoxin/NADPH-dependent thioredoxin reductase C (Trx/NTRC) system mediates this redox switch in planta (Gelhaye et al., 2004; Igamberdiev and Bykova, 2018; Florez-Sarasa et al., 2019). Second, metabolite-dependent allosteric activation: the fully reduced AOX dimer can be further activated via binding of 2-oxo acids to CysI through thiohemiacetal linkage, while CysII and CysIII synergistically modulate this activation process (Millar et al., 1993; Selinski et al., 2016, 2017, 2018a). In *Arabidopsis*, AOX1A displayed markedly higher sensitivity to 2-oxo acid effectors than AOX1C and AOX1D isoforms (Selinski et al., 2016, 2017, 2018a). Systematic screening of TCA cycle intermediates further confirmed widespread isoform-specific differences in AOX metabolite responsiveness, which explained the lack of functional redundancy among distinct AOX paralogs in plant physiological regulation (Selinski et al., 2018a).

Cytoplasmic male sterility (CMS) is an essential agronomic trait that underpins heterosis utilization and hybrid breeding in major crops. Nevertheless, the molecular regulatory mechanisms governing pollen abortion in cotton CMS lines remain largely elusive, severely hindering the identification and functional characterization of fertility restoration genes and limiting the large-scale commercial application of cotton hybrid cultivars (Wang et al., 2010; Zhang et al., 2019). Accumulating studies have demonstrated that CMS is tightly coupled to dysfunction of the mitochondrial electron transport chain (mETC), which involves dysregulation of both COX and AOX respiratory pathways (Sabar et al., 2000; Karpova et al., 2002; Zhou et al., 2019; Du et al., 2019; Li et al., 2019; Hao et al., 2021a, 2021b; Yang et al., 2022; Zhang et al., 2022, 2023a; Li et al., 2022). Notably, altered AOX expression does not act as a direct causal factor for CMS occurrence; instead, it represents a typical adaptive stress response triggered by disrupted cellular redox homeostasis during microspore development. Mitochondrial gene mutations or abnormal modifications frequently impair mETC function, and plants compensate for such respiratory defects by activating alternative respiratory pathways to sustain fundamental metabolic homeostasis (Johns et al., 1993).

Our previous studies had systematically characterized the upland cotton CMS line Jin A, whose pollen abortion phenotype was closely associated with aberrant ROS overaccumulation during anther development (Huang et al., 2004; Zhang et al., 2023a). Further mechanistic evidence revealed that excessive ROS derived from mitochondrial Complex III induced premature tapetal programmed cell death (PCD), accompanied by significantly reduced activities of endogenous ROS-scavenging enzymes (Zhang et al., 2023a). Moreover, transcriptome profiling identified substantial differential expression of *GhAOX* family genes during the pollen abortion process of Jin A (Yang et al., 2014). Collectively, these findings highlight the urgent need to dissect the functional linkage between AOX-mediated redox regulation and ROS homeostasis, as well as its essential role in modulating microspore abortion in cotton CMS lines.

Although the AOX gene family is widely conserved across plant species, a systematic genome-wide identification and comprehensive functional characterization of the *GhAOX* family in upland cotton (*Gossypium hirsutum*) remain unreported to date. *AOX1* is widely recognized as a key stress-responsive regulator, whose transcript abundance is typically induced under various stress conditions. Nevertheless, whether *GhAOX1* also participates in mediating responses to physiological endogenous oxygen stress remains unclear. Similarly, it remains unknown whether *GhAOX* activity can be effectively enhanced under such physiological conditions. Furthermore, it has yet to be determined whether the expression of abortion-associated genes establishes a favorable microenvironment that facilitates ROS scavenging by *GhAOX*. As part of our long-term research program, we performed a comprehensive investigation into the expression patterns and regulatory mechanisms of *GhAOX* genes in the present study. We genome-widely identified *GhAOX* family members from the upland cotton genome and predicted their biological functions. By profiling their expression dynamics during microspore abortion in the CMS line Jin A, we screened *GhAOX* family members that respond to oxygen stress and further explored the transcription factors or regulatory proteins governing their expression. In addition, we analyzed the post-translational modification characteristics of *GhAOX* proteins. Elucidating the functions of *GhAOX* in CMS systems will not only advance our understanding of how abortion-related genes modulate plant metabolic processes but also offer novel perspectives on the roles of AOX in regulating phenotypic traits, stress tolerance, and molecular mechanisms in plants.

## 2 Materials and methods

### 2.1 Plant materials

The plant materials used in this study included the cotton CMS line Jin A and its maintainer line Jin B. Jin A was a CMS line harboring *Gossypium hirsutum* cytoplasm, which was derived from triple interspecific hybrids [(*G. hirsutum*) × (*G. thurberi*)] × [(*G. arboreum*) × (*G. hirsutum*)]. This line was subsequently developed via successive backcrossing using the fertile upland cotton maintainer line Jin B (possessing normal AD1 cytoplasm) as the recurrent parent (Huang et al., 2004; Yang et al., 2014). All cotton plants were cultivated under natural field conditions in Taigu, China, during the conventional growing season. Upon flowering, flower buds at distinct developmental stages were harvested. Based on microscopic observations of microspore development and the abortion characteristics of Jin A reported previously, three developmental stages were classified according to bud transverse diameter (BTD): Stage B (pre-abortion stage, BTD ≤ 1.50 mm), Stage D (abortion-occurring stage, 1.50 < BTD ≤ 2.60 mm), and Stage A (complete abortion stage, BTD > 2.60 mm) (Yang et al., 2014; Zhao et al., 2020). Collected samples from each stage were divided into independent aliquots (0.1~0.2 g) with three biological replicates to guarantee sufficient material for subsequent experiments. All aliquots were immediately snap-frozen in liquid nitrogen and stored at -80°C for downstream molecular analyses.

### 2.2 Identification and bioinformatics analysis of *GhAOX* family members in *G. hirsutum*

The genome sequence and gene annotation files of upland cotton *G. hirsutum* (TM-1 HAU_v1.1) were retrieved from the CottonGen database (https://www.cottongen.org/) as previously reported (Wang et al., 2019; Yu et al., 2014). The protein sequences of *Arabidopsis thaliana* were downloaded from the UniProt database (https://www.uniprot.org). Using *Arabidopsis* AOX protein sequences as query references, a genome-wide screening of cotton AOX homologs was performed via TBtools, with a strict E-value cutoff of 1e-5, NumofHit of 500, and NumofAligns of 250. The conserved functional domains of all candidate *GhAOX* proteins were further verified using the NCBI Conserved Domain Database (CDD, http://www.ncbi.nlm.nih.gov/cdd). Redundant gene and protein sequences were eliminated using CD-HIT with default parameter settings.

Gene structural characteristics (exon-intron organization) and gene duplication events of *GhAOX* family members were analyzed using TBtools. The conserved protein motifs of *GhAOX* were identified via the MEME suite (https://meme-suite.org/meme/tools/meme). The *cis*-acting regulatory elements in the promoter regions of *GhAOX* genes were predicted using the PlantCARE database (http://bioinformatics.psb.ugent.be/webtools/plantcare/html/). Additionally, the open reading frame (ORF) sequences and corresponding protein sequences of candidate *GhAOX* genes were predicted using the NCBI ORF Finder tool.

Public RNA-seq datasets covering eight distinct cotton tissues (bract, petal, torus, root, leaf, stem, pistil, sepal, and anther) and multiple abiotic stress treatments of the TM-1 cultivar were obtained from the NCBI Sequence Read Archive (SRA) under the accession number PRJNA490626 (Sayers et al., 2021). Raw RNA-seq reads were quality-filtered using SOAPnuke v2.1.0 with customized parameters: lowQual = 15, nRate = 0.1, qualRate = 0.5, maxReadLen = 150, and minReadLen = 30, while all other parameters were set to default values. The high-quality clean reads were subsequently aligned to the *G. hirsutum* TM-1 reference genome using Hisat2 with default parameters (Kim et al., 2015; Hu et al., 2019). Transcript abundance of individual genes was quantified and normalized to fragments per kilobase of exon model per million mapped reads (FPKM) using StringTie with default settings (Pertea et al., 2015). Genes with an FPKM value greater than 1 in at least three biological replicates were defined as stably expressed genes. The read counts mapped to each annotated gene were calculated via FeatureCounts for subsequent differential expression analysis (Liao et al., 2014). Differentially expressed genes (DEGs) were identified using DESeq2, with the threshold criteria set as |log2(fold change)| ≥ 1.5 and adjusted *p* < 0.05 (Love et al., 2014). Finally, the expression profiles of *GhAOX* family genes were normalized and compared via log2(FPKM + 1) transformation.

### 2.3 Phylogenetic analysis

The amino acid sequences of *GhAOX* family proteins were retrieved from the UniProt, NCBI, and CottonFGD databases (**Supplementary File 1**). AOX protein sequences from multiple plant species, including *A. thaliana*, maize, and rice (*Oryza sativa*), were collected for comparative analysis. All acquired sequences were aligned using MEGA 7.0 with default pairwise and multiple sequence alignment parameters (**Supplementary File 2**). Based on the alignment results, a phylogenetic tree was constructed using the neighbor-joining algorithm implemented in MEGA 7.0, with the following parameter settings: 1000 bootstrap replicates, Jones-Taylor-Thornton (JTT) substitution model, and gamma-distributed rate variation among sites. The *GhAOX* subfamilies were further classified according to the topological characteristics of the phylogenetic tree and the established AOX classification criteria of other plant species.

### 2.4 RT-PCR and qRT-PCR analysis

Flower bud samples of cotton at different developmental stages were collected to detect the transcript levels of candidate *GhAOX* genes. Total RNA was isolated using the Plant RNA Extraction Kit (Aidlab Biotechnologies Co., Ltd., China) in strict accordance with the manufacturer’s protocols. RNA integrity was verified using 1% agarose gel electrophoresis, which revealed distinct, sharp bands corresponding to 28S and 18S rRNA. The 5S rRNA band was faint and barely detectable. The 28S rRNA band exhibited greater band intensity and width relative to the 18S rRNA band, with no smearing or trailing observed across all samples (Supplemental **Supplementary Figure 1**). The luminance ratio of 28S rRNA to 18S rRNA quantified using ImageJ ranged from 1.83 to 1.97. These results confirmed that the extracted total RNA was of high integrity and quality. The total RNA quality criteria applied for overexpression and gene silencing assays were consistent with those described above. RNA concentrations were quantified by measuring UV absorbance at 260 nm using a NanoPhotometer^®^ N60 (Implen, Germany). The RNA concentrations corresponding to Jin B at Stage B, Jin A at Stage B, Jin B at Stage D, Jin A at Stage D, Jin B at Stage A, and Jin A at Stage A were 498.38, 356.55, 328.80, 451.46, 459.77, and 494.90 ng/μL, respectively. For first-strand cDNA synthesis, reverse transcription was performed using the PrimeScript™ RT reagent Kit with gDNA Eraser (Perfect Real Time, Takara Bio, Dalian, China) to eliminate genomic DNA contamination.

Conventional RT-PCR was carried out using 2 × Taq PCR Master Mix II (TIANGEN Biotech Co., Ltd., Beijing, China) following the manufacturer’s instructions. The PCR amplification program was set as an initial pre-denaturation at 94°C for 3 min, followed by 30 cycles of denaturation at 94°C for 30 s, annealing at 55°C for 30 s, and extension at 72°C for 30 s. Amplified target fragments were separated and verified by 1% agarose gel electrophoresis.

Quantitative real-time PCR (qRT-PCR) assays were performed using TB Green^®^ Premix Ex Taq™ II (Tli RNaseH Plus, Takara Bio, Dalian, China) on a Bio-Rad CFX Connect Real-Time PCR Detection System (Bio-Rad Laboratories, Inc., USA). The qRT-PCR program consisted of an initial denaturation at 94°C for 30 s, followed by 45 cycles of 94°C for 30 s, 55°C for 30 s, and 72°C for 30 s. According to previous studies (Yang et al., 2014; Zhao et al., 2020), *GhEF1α* exhibits stable expression during cotton microspore development and was therefore selected as the internal reference gene for normalization.

Both primer amplification efficiency and qRT-PCR reaction efficiency ranged from 0.9 to 1.0. The amplification curves presented a standard S-shaped profile, and a single sharp peak was observed in the melting curve analysis, confirming the specificity and reliability of the qRT-PCR system. The relative expression levels of target genes were calculated using the 2^-ΔΔCT^ method. All experiments were performed with three independent biological replicates. The full-length sequences of *GhAOX* genes and all primer sequences used in this study were provided in **Supplementary File 3** and Supplemental **Supplementary Table 1**, respectively. Primer synthesis and target gene sequencing were completed by Beijing Tsingke Biology Co., Ltd.

### 2.5 VIGS and overexpression assays

Virus-induced gene silencing (VIGS) was performed using a tobacco rattle virus (TRV)-mediated system to specifically silence *GhAOX2b1/2* and *GhAOX2c1/2* in cotton seedlings (Pang et al., 2013). The target gene fragments spanning 300~500 bp of non-conserved coding regions were amplified and inserted into the TRV2 vector. The recombinant vectors TRV2:*GhAOX2b1/2* and TRV2:*GhAOX2c1/2* were constructed via homologous recombination using the ClonExpress II One Step Cloning Kit (Vazyme Biotech Co., Ltd., Nanjing, China) with *Bam*H I and *Eco*R I restriction enzyme digestion. The validated recombinant constructs were transformed into *Agrobacterium tumefaciens* strain GV3101 and subsequently infiltrated into cotyledons of healthy Jin B seedlings, with a minimum of 30 individual seedlings injected for each treatment. All infiltrated cotton plants were cultivated in a growth chamber under a 16 h light/8 h dark photoperiod at 26°C with a light intensity of 8000 Lx. After 15 days of cultivation, poorly growing seedlings were eliminated, and three independent biological replicates were retained, each containing at least five silenced plants. No obvious morphological differences were observed at the seedling stage between silenced plants and empty-vector control plants. The silencing efficiency of target genes was quantified via qRT-PCR to evaluate the reduction in transcript abundance. The contents of superoxide anion and hydrogen peroxide (H_2_O_2_) were determined following the protocol described by Zhang et al. (2023a). All VIGS-related experiments were performed in three independent replicates.

For gene overexpression assays, the full-length ORFs of *GhAOX1a1/2* and *GhAOX2a1/2-c1/2* were separately cloned into the pRI101-AN expression vector. Vector construction was conducted using *Nde* I and *Bam*H I restriction digestion combined with homologous recombination via the ClonExpress II One Step Cloning Kit (Vazyme Biotech Co., Ltd., Nanjing, China). *A. thaliana* Columbia-0 wild-type (wt) plants were transformed using the floral dip method according to a previously established protocol (Clough and Bent, 1998). At least three independent transgenic lines were generated and screened for subsequent phenotypic and molecular identification. *Arabidopsis* plants were grown under long-day conditions (16 h light/8 h dark) with white fluorescent illumination, with daytime and nighttime temperatures set at 23°C and 21°C, respectively, and a relative humidity of 60%. Transgenic lines with significantly elevated *GhAOX* expression levels were selected for further functional characterization. The developmental stages of *Arabidopsis* stamens were classified based on the previously reported staging standard (Sanders et al., 1999). NBT and DAB histochemical staining, quantitative detection of superoxide anion and H_2_O_2_, and microscopic observation of stamen microstructure were performed in accordance with the method described by Zhang et al. (2023a). All overexpression experiments were repeated three times independently.

Fresh mature pollen grains collected from transgenic and wt *Arabidopsis* plants were used for *in vitro* pollen germination assays. Pollen grains were evenly spread on solid germination medium containing 1 mM Ca(NO_3_)_2_, 1 mM CaCl_2_, 1 mM MgSO_4_, 1 mM H_3_BO_3_, 18% sucrose, and 0.6% agar. The cultured samples were incubated in a humid chamber at 25°C under dark conditions for more than 8 h, and pollen germination status was observed and photographed using an optical microscope. The pollen germination experiments were conducted with three independent biological replicates.

### 2.6 Yeast one-hybrid assay

A approximately 300 bp promoter fragment of *GhAOX2c1/2* was amplified and inserted into the pAbAi bait vector for Y1H screening. The recombinant bait vector was constructed via homologous recombination using the ClonExpress II One Step Cloning Kit (Vazyme Biotech Co., Ltd., Nanjing, China) with *Hind* III and *Xho* I restriction enzyme digestion. The successfully constructed *GhAOX2c1/2*-AbAi vector was transformed into yeast Y1HGold competent cells, and positive yeast strains were preliminarily screened on synthetic dropout medium lacking uracil (SD/-Ura). Self-activation verification demonstrated that 100 ng/mL aureobasidin A (AbA) was sufficient to suppress the basal growth of *GhAOX2c1/2*-AbAi yeast on SD/-Ura medium. To eliminate false-positive interactions and improve screening stringency, the AbA concentration was increased to 200 ng/mL for subsequent library screening.

A cDNA library was constructed using cotton anther tissues of Jin A at Stage D. The cDNA library were co-transformed with the pGADT7-Rec prey vector into *GhAOX2c1/2*-AbAi yeast strains. Transformants were cultivated on high-stringency selective medium containing 200 ng/mL AbA (SD/-Ura/-Leu + 200 ng/mL AbA). Yeast colonies that grew normally on the selective medium were regarded as positive interactive clones. Plasmids were extracted from these positive colonies and subjected to Sanger sequencing. The validated candidate sequences were individually recombined into the pGADT7 vector, and pairwise Y1H verification was performed on SD/-Ura/-Leu + 200 ng/mL AbA medium to confirm authentic interactions.

Sequences of candidate interactive genes were aligned using NCBI BLAST (https://blast.ncbi.nlm.nih.gov/Blast.cgi), and the corresponding protein functional information was retrieved from the UniProt database (https://www.uniprot.org/). Gene Ontology (GO) functional enrichment analysis was performed using the online Omicshare tools (https://www.omicshare.com/tools).

Transcript expression profiles of candidate genes during the microspore abortion stage of Jin A were obtained from a previously published RNA-seq dataset (Yang et al., 2014), which is available in the NCBI SRA under the accession numbers SRX547770, SRX547777, SRX547779, and SRX547781. Data processing was the same as the previous description.

### 2.7 Western blotting and AOX enzymatic activity assay

Western blotting (WB) analysis was performed following a previously described protocol (Taylor and Posch, 2014). A 13% sodium dodecyl sulfate-polyacrylamide gel electrophoresis (SDS-PAGE) gel was used for total protein separation. The endogenous *GhAOX* protein was detected using a rabbit anti-AOX1/2 polyclonal antibody (Agrisera), and anti-plant actin antibody (Abbkine) was employed as the internal loading control for protein normalization. For immunoblotting, the primary antibodies (anti-AOX1/2 and anti-plant actin) and secondary anti-rabbit IgG antibody were diluted at 1:1000 and 1:10000, respectively. All WB experiments were conducted with three independent biological replicates, and consistent banding patterns were observed across replicates. The clearest and most representative immunoblot bands were selected for subsequent quantitative analysis. Band grayscale intensity was quantified using ImageJ software to determine the relative protein abundance.

The enzymatic activity of AOX was measured using a commercial alternative oxidase activity detection kit (Jiangsu Meimian Industrial Co., Ltd., China) in accordance with the manufacturer’s standard protocols. Briefly, approximately 0.1 g of fresh cotton anther samples was homogenized in phosphate-buffered saline (PBS) for protein extraction. The prepared samples were loaded into detection wells, followed by plate washing and chromogenic reaction. The absorbance value at 450 nm was subsequently measured to calculate AOX enzymatic activity. All assays were performed with three independent biological replicates to ensure experimental reliability.

### 2.8 Prokaryotic expression, purification and *GhAOX* activation assay

The *E. coli* BL21 prokaryotic expression system was utilized for recombinant *GhAOX* protein purification according to a previously established protocol (Sankar et al., 2022). The full-length *GhAOX* coding sequences were subcloned into the pET-22b(+) expression vector using *Hind* III and *Bam*H I restriction enzymes, with vector construction procedures consistent with the cloning methods described above. Recombinant plasmids were transformed into *E. coli* Transetta (DE3) competent cells for heterologous protein expression, and detailed protein induction conditions are listed in Supplemental **Supplementary Table 2**. The recombinant His-tagged *GhAOX* protein was purified using an ÄKTA pure protein purification system (GE HealthCare), which enables high-efficiency protein separation and purification via precise control of flow rate, elution gradient, and detection wavelength.

Protein purification was performed based on the specific binding affinity between the His-tag and metal ions. The imidazole groups of histidine residues on the recombinant protein coordinate with transition metal ions (e.g., Ni and Co), allowing specific adsorption to metal affinity chromatography media. Bound target proteins were eluted with imidazole solutions of optimized concentrations. The molecular weight of the purified recombinant *GhAOX* protein was consistent with the theoretical prediction (Supplemental **Supplementary Figure 2**). The concentration of purified protein samples was quantified using the Micro BCA Protein Assay Kit (Boster Biological Technology Co., Ltd., China).

The TCA cycle intermediates used for *GhAOX* activation assays, including pyruvate, citrate, isocitrate, 2-oxoglutarate (2-OG), succinate, fumarate, malate, and oxaloacetate (OAA), were prepared following the method described by Selinski et al. (2018a). All intermediates were configured as 1 M stock solutions, with pH adjusted to 7.0~7.5, and diluted to a final working concentration of 5 mM prior to use.

*GhAOX* activity assays in response to TCA intermediates were performed according to a previously reported method (Li et al., 2012). The substrate tetramethylhydroquinone was used for enzymatic reaction detection, which exhibits a characteristic absorption peak at 259 nm with an extinction coefficient of 17.8 L·mmol^-1^·cm^-1^. Enzyme activity was calculated based on the Beer-Lambert law. For standardized quantification, one unit of *GhAOX* activity was defined as a 1.0 absorbance change per milligram of purified protein within 1.5 min of reaction.

Briefly, the reaction system contained 0.25 mM tetramethylhydroquinone dissolved in 80 mM KH_2_PO_4_-KOH buffer (pH 7.0~7.5). After the addition of 5 mM individual TCA intermediates and 50 μL purified *GhAOX* protein, the absorbance at 259 nm was continuously monitored within 2 min. Reaction mixtures without purified *GhAOX* protein were set as blank controls. The relative *GhAOX* activity was calculated using the formula: Ra = [(OD2 - OD1) - (A2 - A1)]/(*c* × 0.05), where OD1 and OD2 represent the absorbance values at 10 s and 100 s after the addition of all reaction components, respectively; A2 and A1 indicate the absorbance values of blank control groups at 10 s and 100 s; *c* denotes the protein concentration (mg/mL) of purified *GhAOX*. All enzyme activity data were normalized relative to the basal activity measured in the absence of TCA intermediate effectors. Each assay was conducted with three independent biological replicates.

Plant mitochondria were isolated from cotton vegetative tissues and anthers using the Plant Mitochondrial Extraction Kit (Beijing Biolab Biotechnology, https://www.bjbalb.com/) following the manufacturer’s protocols. Briefly, approximately 2 g of fresh plant samples were ground in pre-cooled grinding buffer, and the entire extraction procedure was performed at 4°C to maintain mitochondrial activity and integrity. Cell debris and plastids were removed via low-speed centrifugation, and crude mitochondrial pellets were subsequently harvested by high-speed density gradient centrifugation.

The endogenous contents of total and mitochondrial pyruvate, 2-OG, and OAA were determined using commercial detection kits (Beijing Solarbio Science and Technology Co., Ltd. and Jiangsu Meimian Industrial Co., Ltd., China) in accordance with the corresponding manufacturer’s instructions. All quantification experiments were repeated three times independently to ensure data reproducibility.

### 2.9 Statistical methods

Differences between two groups were assessed using Student’s *t*-test in Excel.

## 3 Results

### 3.1 Phenotypic characteristics of CMS line Jin A anthers (pollen grains)

Compared with the maintainer line Jin B, the CMS line Jin A exhibited defective floral phenotypes, including shortened filaments and smaller anthers (Supplemental **Supplementary Figure 3a**). Alexander and I_2_-KI staining assays revealed that Jin B produced morphologically normal, spherical pollen grains with typical needle-like surface ornaments, which were stained deep red and brown, respectively. In contrast, no viable pollen grains were detected in the anthers of Jin A (Supplemental **Supplementary Figure 3b**).

### 3.2 Identification and classification of *GhAOX* family members

**Figure 1:**
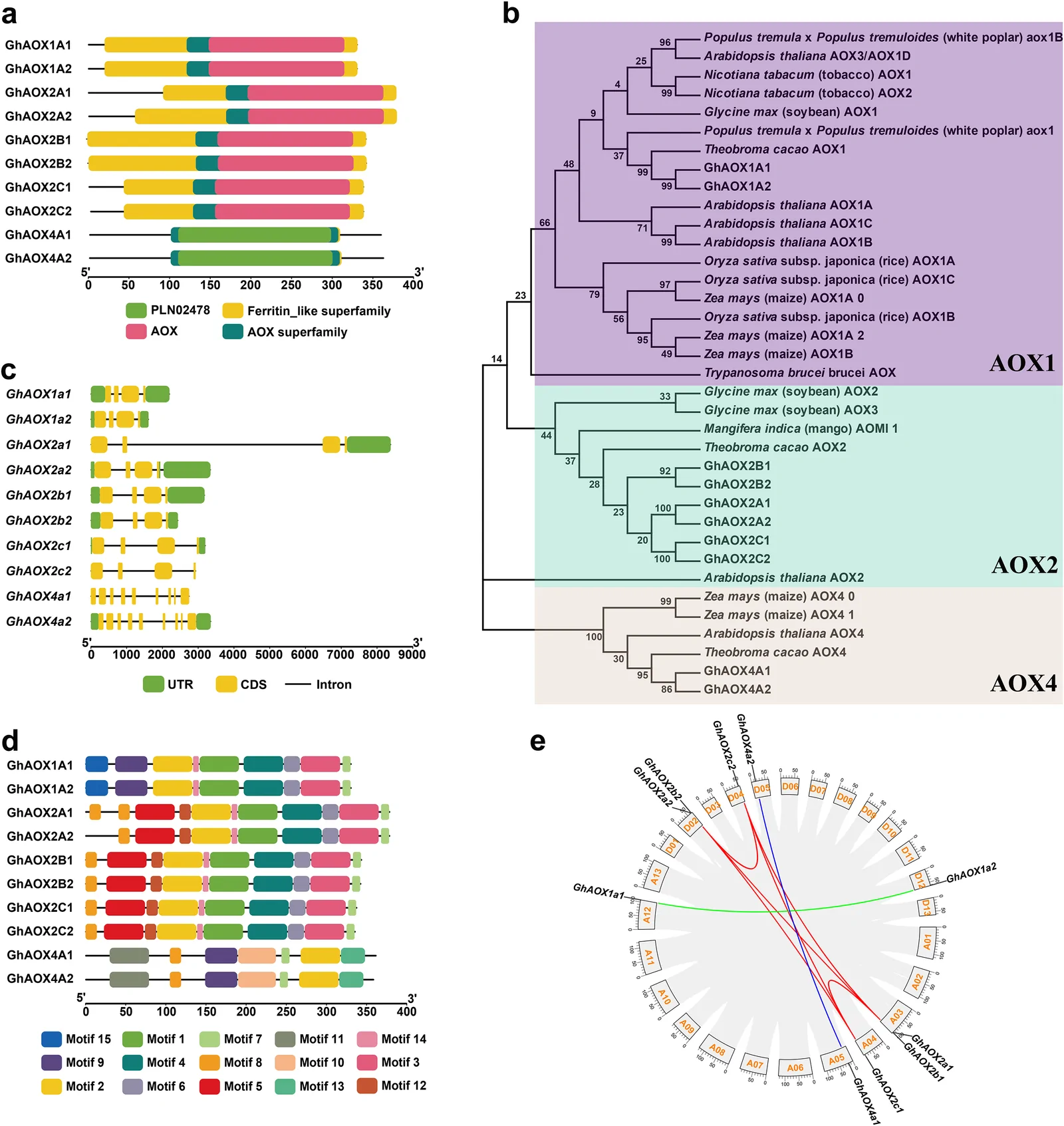
Bioinformatic characterization of *Gossypium hirsutum AOX* gene family. **(a)** Conserved domain verification of *GhAOX* proteins via the NCBI Conserved Domain Database (CDD). All *GhAOX* sequences harbored intact Ferritin_like and AOX superfamily conserved domains. **(b)** Phylogenetic analysis of AOX proteins from *G. hirsutum* and other plant species. A total of 36 AOX protein sequences were used to construct the maximum likelihood (ML) tree in MEGA 7.0, with 1, 000 bootstrap replicates for statistical reliability evaluation. **(c)** Exon-intron structural organization of the *GhAOX* family genes. **(d)** Conserved motif distribution of *GhAOX* proteins. Fifteen distinct conserved motifs were identified and annotated with different colored boxes. **(e)** Chromosomal distribution and paralogous gene pairs of the *GhAOX* family. The scale bar represents chromosome length in megabases (Mbp).

The *A. thaliana AOX* gene family consists of five canonical members, namely *AtAOX1a*-*d* and *AtAOX2* (Polidoros et al., 2009). To systematically identify *GhAOX* family members in upland cotton, BLASTP searches were performed against the *G. hirsutum* TM-1 reference genome, using the five *Arabidopsis* AOX proteins (AtAOX1A-D and AtAOX2) and their homologous protein AtAOX4/AtPTOX as query sequences. All candidate sequences were further verified via the NCBI Conserved Domain Database (CDD) to confirm the presence of complete and intact AOX conserved domains (**Figure 1a**). After eliminating redundant sequences, a total of 10 non-redundant *GhAOX* genes were ultimately identified in the upland cotton genome, including *Ghir_A12G028850* (*GhAOX1a1*), *Ghir_D12G029000* (*GhAOX1a2*), *Ghir_A03G019170* (*GhAOX2a1*), *Ghir_D02G020510* (*GhAOX2a2*), *Ghir_A03G019180* (*GhAOX2b1*), *Ghir_D02G020520* (*GhAOX2b2*), *Ghir_A04G012310* (*GhAOX2c1*), *Ghir_D04G016730* (*GhAOX2c2*), *Ghir_A05G017800* (*GhAOX4a1*), and *Ghir_D05G017790* (**GhAOX*4a2*) (**Table 1**).

**Table 1:** Basic information of the *GhAOX* family in *Gossypium hirsutum*.

Name	Cotton library ID	NCBI Peference	ID	mRNA length (bp)	Exon number	Intron number	CDS (bp)	Amino acid length (AA)	Groups
*GhAOX1a1*	*Ghir_A12G028850*	NM_001327426.1	LOC107939301	1298	4	3	996	331	*GhAOX1a1/2*
*GhAOX1a2*	*Ghir_D12G029000*	XM_016873154.2	LOC107939782	1448	4	3	996	331	
*GhAOX2a1*	*Ghir_A03G019170*	XM_016812315.2	LOC107888202	1316	4	3	1017	338	*GhAOX2a1/2*
*GhAOX2a2*	*Ghir_D02G020510*	XM_016836286.2	LOC107908964	1335	5	4	1017	338	
*GhAOX2b1*	*Ghir_A03G019180*	XM_016811709.2	LOC107887447	1414	4	3	1038	345	*GhAOX2b1/2*
*GhAOX2b2*	*Ghir_D02G020520*	XM_016836285.2	LOC107908962	1360	4	3	1035	344	
*GhAOX2c1*	*Ghir_A04G012310*	XM_016883862.2	LOC107949133	1256	4	3	1020	339	*GhAOX2c1/2*
*GhAOX2c2*	*Ghir_D04G016730*	XM_041090909.1	LOC121215954	1597	4	3	1014	337	
*GhAOX4a1*	*Ghir_A05G017800*	XM_016832372.2	LOC107905673	1565	9	8	1089	362	*GhAOX4a1/2*
*GhAOX4a2*	*Ghir_D05G017790*	XM_016832680.2	LOC107905897	1637	9	8	1080	359	

Given the heterotetraploid nature of upland cotton, homologous sequences derived from the A and D subgenomes were not annotated separately. Instead, subgenome origins were distinguished via terminal numerical labels: the suffix “1” denotes sequences from the A subgenome, whereas the suffix “2” indicates sequences from the D subgenome.

To clarify the phylogenetic classification and evolutionary relationships of cotton AOX proteins, a maximum likelihood (ML) phylogenetic tree was constructed using AOX and AOX4/PTOX protein sequences from multiple plant species, including maize, rice, and cocoa. The AOX sequence of *Trypanosoma brucei* brucei was also incorporated for the analysis of conserved amino acid residues, structural characteristics, and evolutionary modeling, and all protein sequences were retrieved from the UniProt database (**Supplementary File 1**). Phylogenetic analysis categorized all AOX proteins into three distinct clades (**Figure 1b**; **Supplementary File 1**). Clade I comprised AOX1 subfamily members from both monocot and dicot plant species, including two upland cotton isoforms (*GhAOX1A1* and *GhAOX*1A2), as well as *Trypanosoma* AOX proteins. Clade II exclusively contained dicot-specific AOX2 subfamily proteins derived from soybean, mango, cocoa, and *Arabidopsis*, covering six *GhAOX* members identified in upland cotton. Clade III corresponded to the chloroplast-localized AOX4/PTOX subfamily, which included homologs from maize, *Arabidopsis*, cocoa, and two upland cotton proteins (*GhAOX4A1* and *GhAOX4A2*).

The nomenclature of all *GhAOX* genes was standardized according to their phylogenetic clustering, sequence homology, and chromosomal distribution, with clear subgroup definitions provided in **Table 1**. Notably, the naming system established in the present study is specific to this research. Furthermore, phylogenetic topology revealed that all *GhAOX* members clustered closely with their cocoa homologs, indicating a relatively close evolutionary relationship between upland cotton and cocoa AOX family genes.

### 3.3 Gene structure and conserved motif analysis of *GhAOX* family members

To explore the structural evolutionary characteristics of *GhAOX* family genes, the exon-intron organization of all identified *GhAOX* members was systematically analyzed (**Figure 1c**). Gene structure analysis showed that *GhAOX1a1/2*, *GhAOX2a1*, *GhAOX2b1/2*, and *GhAOX2c1/2* possessed a conserved gene structure consisting of four exons and three introns, which is consistent with the typical structural characteristics of *AOX* genes in other plant species and reflects the high evolutionary conservation of plant *AOX* families. Differentially, an intron was inserted within the downstream UTR region of *GhAOX2a2*. In addition, the two *GhAOX4* subfamily genes (*GhAOX4a1/2*) exhibited a distinct structural pattern with nine exons and eight introns, which was markedly different from that of the *GhAOX1* and *GhAOX2* subfamilies (**Figure 1c**).

Conserved protein motif analysis of *GhAOX* proteins was subsequently performed using the MEME suite to characterize the structural conservation and divergence among family members (**Figure 1d**; Supplemental **Supplementary Figure 4**). Homologous *GhAOX* proteins derived from the A and D subgenomes of upland cotton exhibited highly consistent motif compositions, further verifying their close genetic homology. Multiple core motifs (motif-2, motif-14, motif-1, motif-4, motif-6, motif-3, and motif-7) were sequentially distributed at the C-terminus of *GhAOX1* and *GhAOX2* proteins, representing highly conserved structural features among mitochondrial AOX members. In contrast, subfamily-specific variations were predominantly observed in the variable N-terminal regions. Specifically, the *GhAOX1A1/2* proteins were uniquely characterized by the presence of motif-15 and motif-9, while *GhAOX2A1/2* specifically contained motif-8, motif-5, and motif-12. The chloroplast-localized *GhAOX4A1/2* proteins displayed obviously divergent motif arrangements compared with mitochondrial *GhAOX* members. Nevertheless, motif-7 and motif-2 were universally conserved across all three *GhAOX* subfamilies, indicating their essential roles in maintaining the basic biological functions of cotton AOX proteins.

### 3.4 Gene duplication analysis of *GhAOX* family members

To elucidate the evolutionary expansion mechanism of the *GhAOX* gene family, gene duplication events were systematically identified via BLASTP searches and MCScanX analysis. A total of eight paralogous gene pairs were detected in the *G. hirsutum* genome, whereas no tandem duplication events were identified (**Figure 1e**). These results indicate that the expansion of the *GhAOX* gene family is predominantly driven by segmental duplication events. The Ka/Ks ratio is a reliable indicator for evaluating gene selective pressure during evolution. In this study, Ka/Ks values of all eight duplicated *GhAOX* gene pairs were less than 1 (Supplemental **Supplementary Table 3**), demonstrating that these gene pairs have undergone strong purifying selection during cotton evolution, which contributes to the functional conservation of *GhAOX* family genes.

### 3.5 Spatiotemporal and stress-induced expression patterns of *GhAOX* family genes

**Figure 2:**
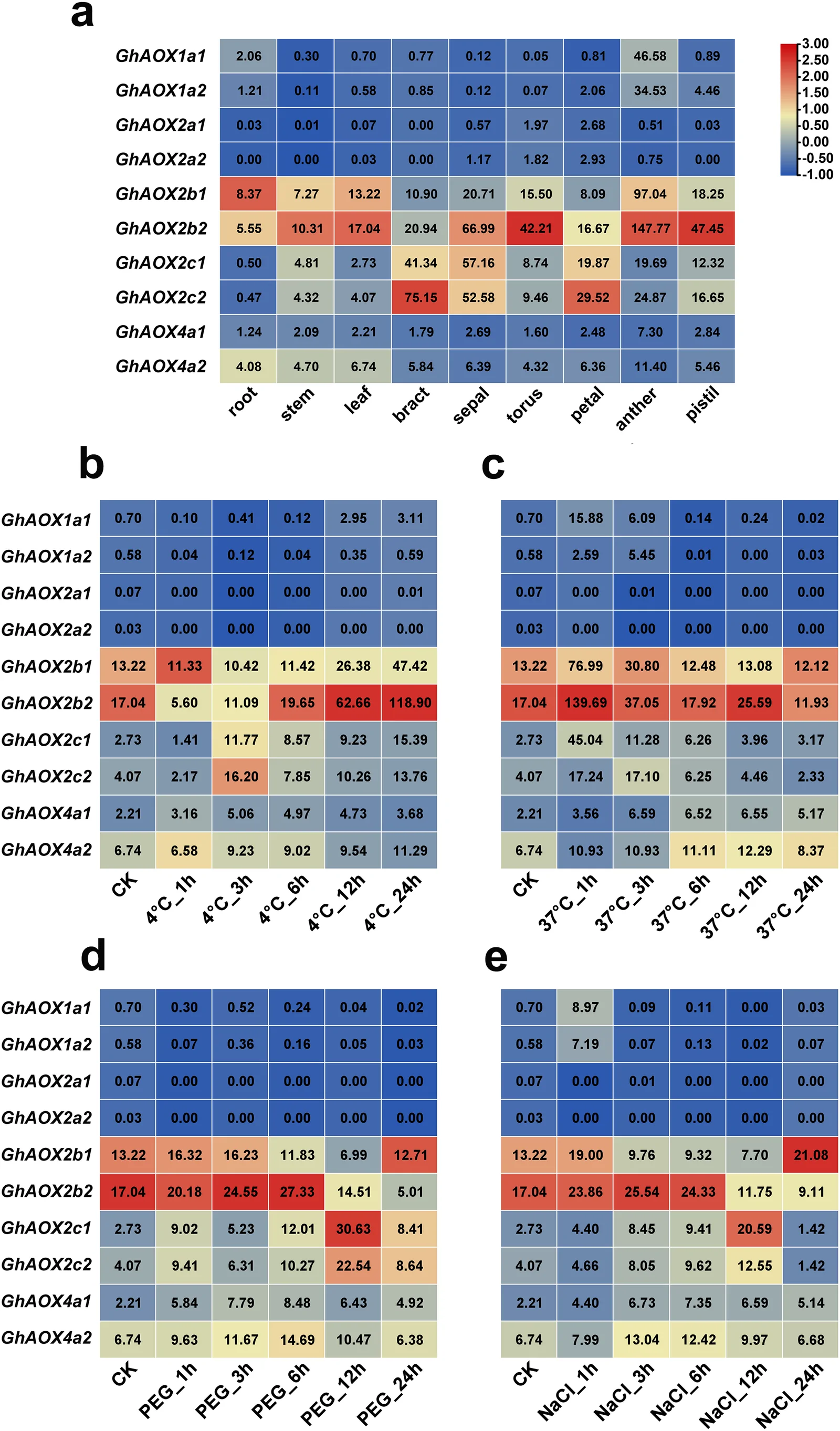
Tissue-specific and stress-responsive expression patterns of *GhAOX* genes in *G. hirsutum*. **(a)** Transcript profiles of *GhAOX* family genes across nine representative cotton tissues, including bract, petal, torus, root, leaf, stem, pistil, sepal, and anther. **(b–e)** Dynamic expression patterns of *GhAOX* genes under four abiotic stress conditions: cold (4℃), heat (37℃), PEG-simulated drought, and NaCl-induced salt stress. All expression data were retrieved from public cotton RNA-seq datasets (NCBI BioProject accession: PRJNA490626; Sayers et al., 2021). Values within rectangular boxes denote the average FPKM derived from three independent biological replicates. The expression profiles of *GhAOX* family genes were normalized and compared via log2(FPKM + 1) transformation. The color gradient scale was standardized and visualized with TBtools software.

To explore the potential biological functions of *GhAOX* genes in cotton growth and development, the tissue-specific expression profiles of all *GhAOX* members were analyzed based on public RNA-seq datasets covering nine distinct cotton tissues, including bract, petal, receptacle, root, leaf, stem, pistil, sepal, and anther (**Figure 2a**). Transcriptome analysis revealed distinct tissue-specific expression patterns and variable expression abundances among *GhAOX* subfamily members. Briefly, *GhAOX1a1/2* were predominantly expressed in stamens, while *GhAOX2a1/2* exhibited higher transcript accumulation in petals and receptacles. *GhAOX2b1/2* were ubiquitously expressed across all detected tissues, with preferential expression in stamens and pistils. *GhAOX2c1/2* displayed floral organ-specific expression, with relatively low transcript levels in roots and leaves. Notably, *GhAOX2b1/2* and *GhAOX2c1/2* represented the most highly expressed *GhAOX* members across multiple tissues, and *GhAOX1a1/2*, *GhAOX2b1/2*, and *GhAOX2c1/2* showed prominent anther-enriched expression, implying their potential roles in cotton floral and anther development.

To further clarify the stress-responsive characteristics of *GhAOX* family genes, their dynamic expression patterns under four typical abiotic stresses (PEG-induced osmotic stress, NaCl-induced salt stress, 4°C cold stress, and 37°C heat stress) were analyzed using published RNA-seq data (**Figures 2b-e**). Subfamily members exhibited divergent expression responses to different stress treatments. *GhAOX1a1/2* were significantly induced at 1 h after NaCl and high-temperature (37°C) treatments. In contrast, *GhAOX2a1/2* maintained constitutively low expression levels and showed negligible transcriptional changes under all four stress conditions, indicating insensitivity to abiotic stress stimuli. The expression of *GhAOX2b1/2* was slightly altered within the first 6 h of PEG and NaCl treatments, with no obvious stress response at the early stage; however, these genes were markedly responsive to extreme temperature stress, reaching peak expression at 24 h under cold treatment and 1 h under heat treatment, respectively. For *GhAOX2c1/2*, transcript abundance increased gradually with prolonged PEG and NaCl treatment, with the highest expression levels observed at 12 h. Moreover, *GhAOX2c1/2* exhibited similar temperature response patterns to *GhAOX2b1/2* under cold and heat stress.

Collectively, these results demonstrate that *GhAOX2b1/2* and *GhAOX2c1/2* are the primary functional members of the *GhAOX* family involved in cotton abiotic stress tolerance. In particular, *GhAOX2c1/2* displayed significant and widespread transcriptional fluctuations in response to osmotic, salt, cold, and heat stresses, suggesting its critical role in mediating cotton stress adaptation.

### 3.6 Promoter cis-acting element analysis of *GhAOX* family members

To uncover the regulatory mechanisms governing *GhAOX* gene expression, promoter sequences of all *GhAOX* family members were cloned and comparatively analyzed between Jin A and Jin B (Supplemental **Supplementary Figure 5**). A large number of cis-acting regulatory elements were identified across these promoter regions. Notably, sequence comparison revealed high conservation of promoter profiles between the two cotton lines, with only three differential cis-elements (AE-box, TCA-element, and W box) detected.

Statistical classification of all predicted cis-elements (175 in total) showed that hormone-responsive elements constituted the largest functional category (67/175), covering abscisic acid (ABA, 28/175), auxin (6/175), ethylene (18/175), and methyl jasmonate (14/175) response motifs. ABA-responsive elements were predominantly enriched in the promoters of *GhAOX1a1/2* (6 elements), *GhAOX2b1/2* (16 elements), and *GhAOX2c1/2* (6 elements). Auxin-related motifs were exclusively distributed in *GhAOX2b1/2* (2 elements) and *GhAOX2c1/2* (4 elements), whereas ethylene-responsive elements were mainly detected in *GhAOX1a1/2* (4 elements) and *GhAOX2c1/2* (14 elements). The second-largest category corresponded to light-responsive elements (47/175), including AE-box, G-box, and GT1-motif. In addition, multiple functional cis-elements associated with flavonoid biosynthesis, mechanical damage response, defense and stress tolerance, and anaerobic adaptation were identified, along with binding sites for key transcription factors such as MYB, WRKY, and stress-responsive STRE motifs.

Notably, *GhAOX2b1/2* and *GhAOX2c1/2* possessed a higher density of functional cis-regulatory elements than *GhAOX1a1/2* and *GhAOX2a1/2*, which provides a plausible molecular explanation for their stronger transcriptional responsiveness to abiotic stress. These results indicate that *GhAOX* genes act as downstream regulatory components in plant hormone signaling and light response pathways. Subfamily-specific variations in cis-element composition further contribute to the differential sensitivity of distinct *GhAOX* isoforms to environmental stimuli in cotton.

### 3.7 Homologous cloning and bioinformatic characterization of *GhAOX* family members

The full-length coding sequences (CDSs) of *GhAOX* subfamily members were successfully cloned from Jin A and Jin B (Supplemental **Supplementary Figure 6**; **Supplementary File 3**). The CDS lengths were 996 bp for *GhAOX1a1/2*, 1017 bp for *GhAOX2a1/2*, 1035 bp for *GhAOX2b1/2*, and 1017 bp for *GhAOX2c1/2*, respectively. Within the Jin A background, the CDS sequence similarity among all *GhAOX* members exceeded 82%. Comparative sequence alignment between the two cotton lines revealed high genetic conservation: the CDS similarity of *GhAOX1a1/2*, *GhAOX2a1/2*, *GhAOX2b1/2*, and *GhAOX2c1/2* reached 99.60%, 98.23%, 97.29%, and 100%, respectively.

**Figure 3:**
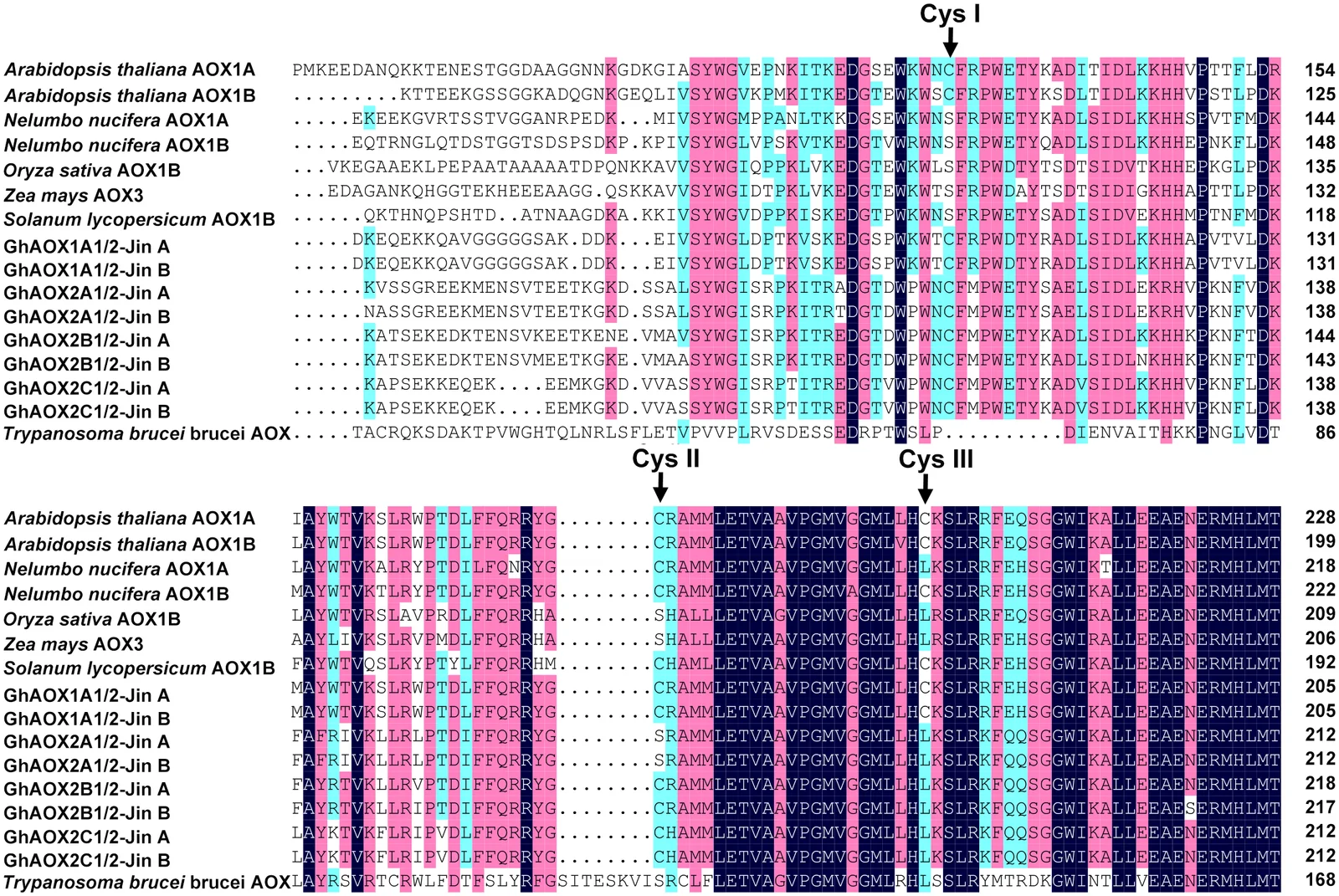
Partial sequence alignment of AOX proteins from cotton and other plant species. Black shading indicates identical amino acid residues, while similar amino acid residues are shaded in pink and blue. Three conserved cysteine residues (CysI, CysII, and CysIII) critical for AOX redox regulation and metabolite activation are specifically labeled.

Subsequent protein sequence prediction was performed using the NCBI ORF Finder tool (**Figure 3**; Supplemental **Supplementary Figure 7**; **Supplementary File 3**). The deduced proteins encoded by *GhAOX1a1/2*, *GhAOX2a1/2*, *GhAOX2b1/2*, and *GhAOX2c1/2* in Jin A contained 331, 338, 344, and 338 amino acid residues, respectively. The overall protein sequence similarity across the four *GhAOX* isoforms was greater than 80%. Cross-line comparison showed that the protein sequence similarity between Jin A and Jin B was 100% for *GhAOX*1A1/2 and *GhAOX*2C1/2, 96.75% for *GhAOX*2A1/2, 96.22% for *GhAOX*2B1/2.

Based on the well-characterized structural features of *Trypanosoma brucei* AOX and previously reported functional residues (Andersson and Nordlund, 1999), we systematically annotated the conserved functional domains and key amino acid sites of cotton *GhAOX* proteins, including two canonical EXXH motifs, iron-coordinating glutamate and histidine residues, ubiquinone-binding sites, dioxygen-binding sites, and three conserved cysteine residues (CysI, CysII, and CysIII). No variations in these core conserved residues and functional domains were detected between Jin A and Jin B.

Nevertheless, prominent isoform-specific variations were observed among different *GhAOX* members. *GhAOX*2A1/2 harbored a non-synonymous substitution at a conserved histidine residue (H→R). *GhAOX*2C1/2 exhibited unique sequence variations in the helical region responsible for constructing the hydroxo-bridged binuclear iron center and in core iron-binding motifs. Additionally, site-specific mutations were identified in the conserved cysteine residues across isoforms: CysI was strictly conserved in all *GhAOX* proteins, while CysII was substituted by serine in *GhAOX*2A1/2. CysIII was exclusively present in *GhAOX*1A1/2 and replaced by leucine in all other *GhAOX* isoforms. Moreover, extensive sequence variations were detected in non-conserved regions outside the functional active centers among the four *GhAOX* subfamily members.

### 3.8 Expression profiling of *GhAOX* family genes during anther development in Jin A

**Figure 4:**
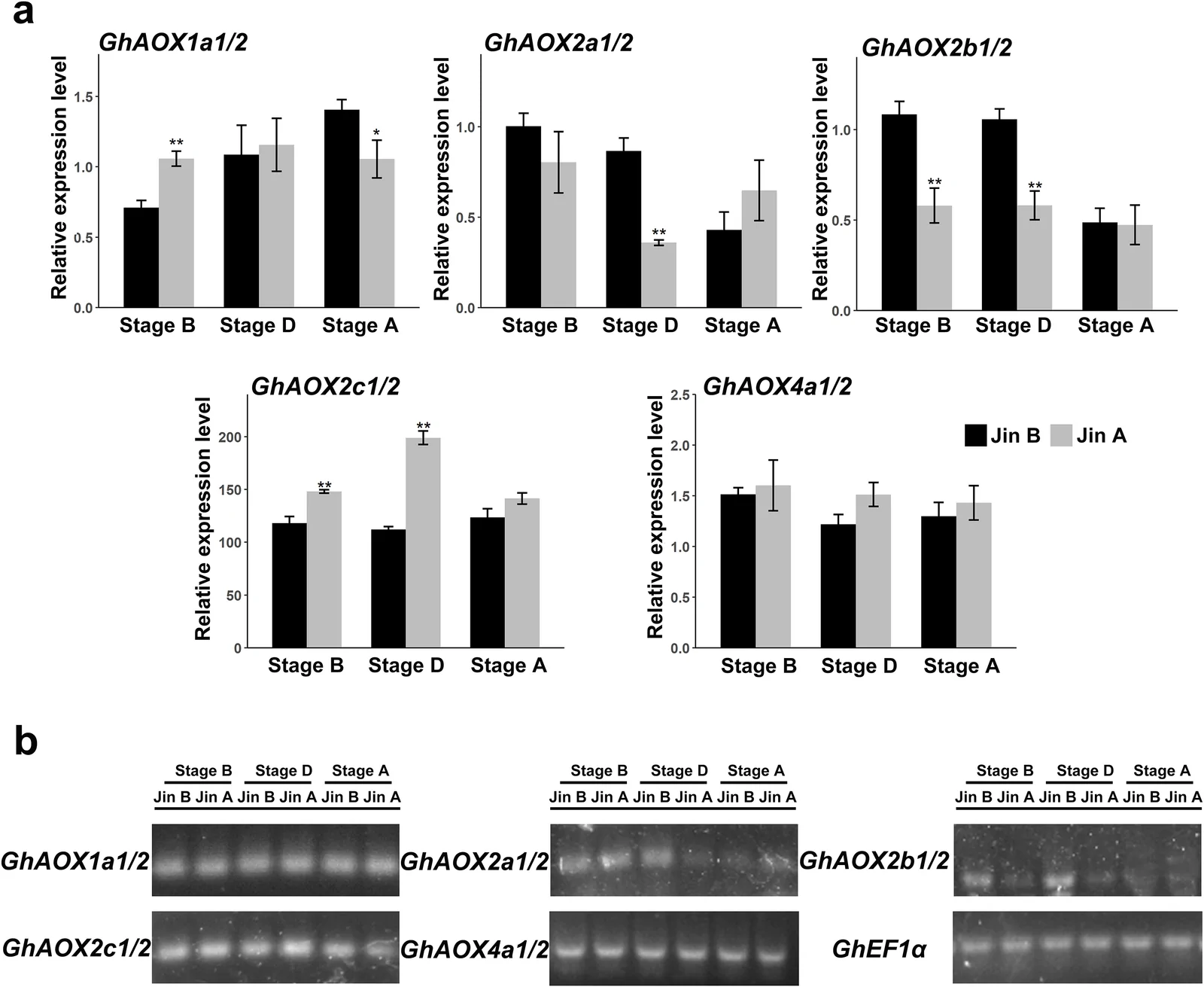
Temporal expression patterns of *GhAOX* family genes during anther development in Jin A and its maintainer Jin B. **(a)** qRT-PCR and **(b)** RT-PCR analyses reveal distinct transcriptional profiles of *GhAOX* members throughout anther developmental stages. The cotton *GhEF1α* gene was used as an internal reference for normalization. Data are presented as means ± SD from three independent experiments. Asterisks indicate statistically significant differences between Jin A and its maintainer Jin B (*P < 0.05, **P < 0.01; Student’s *t*-test).

The regulation of transcription levels is one of the key factors in *GhAOX* regulation. To further characterize the differential expression patterns of *GhAOX* family members during anther development, qRT-PCR analysis was performed to compare transcript abundances between Jin A and Jin B (**Figures 4a, b**). The results revealed distinct expression profiles of *GhAOX* subfamily genes during the microspore abortion process. No significant expression differences in *GhAOX1a1/2* and *GhAOX4a1/2* were detected between Jin A and Jin B throughout anther development. In contrast, the transcript levels of *GhAOX2a1/2* and *GhAOX2b1/2* were significantly downregulated in Jin A at Stage D, relative to Jin B. Notably, *GhAOX2c1/2* exhibited markedly higher expression in Jin A than in Jin B, where its transcript accumulation was approximately two-fold greater than that in the maintainer line.

### 3.9 *GhAOX* functional validation of via heterologous overexpression in *A. thaliana* and VIGS-mediated gene silencing in cotton

**Figure 5:**
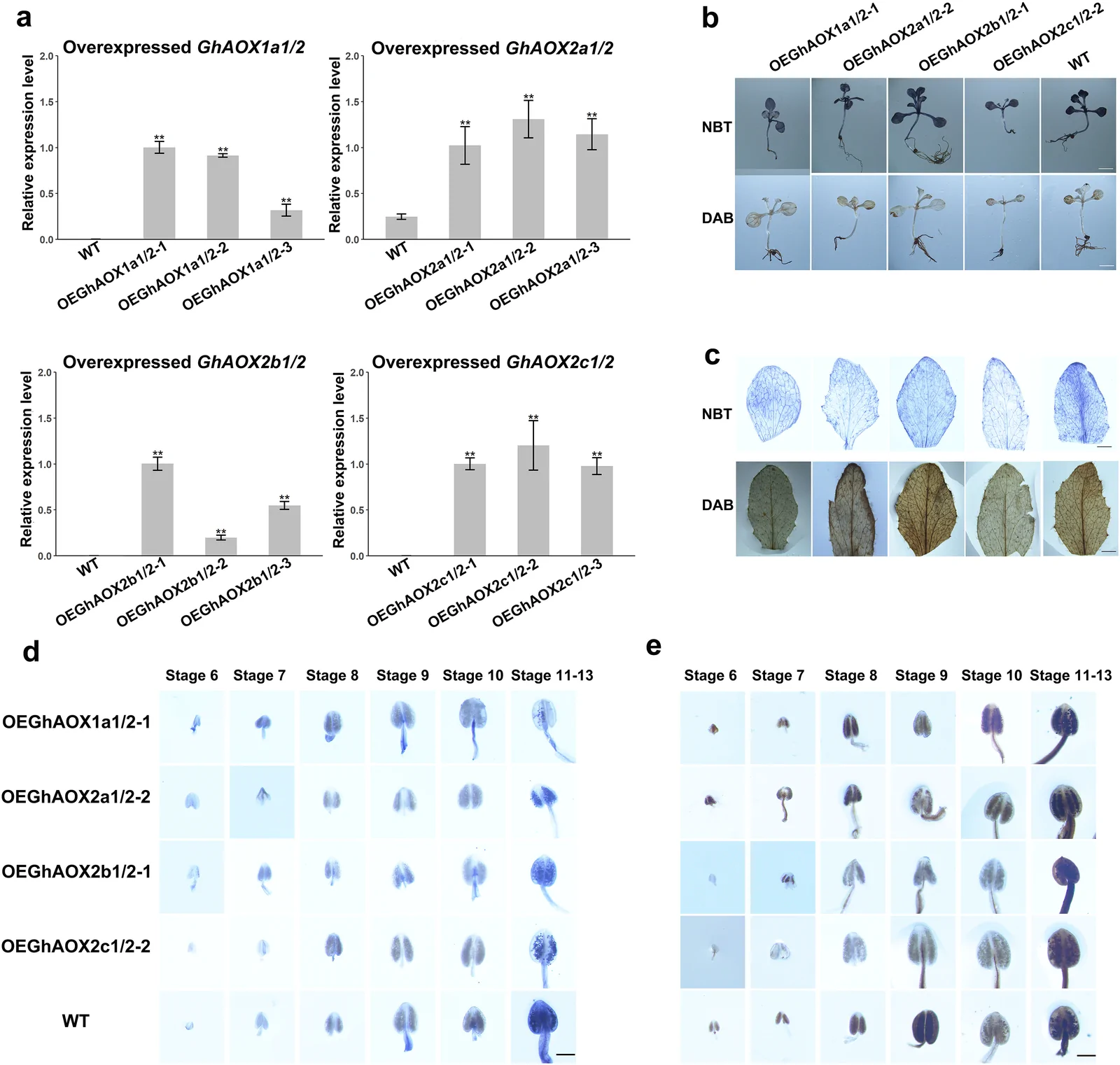
Ectopic overexpression of Jin A *GhAOX* genes in *Arabidopsis thaliana*. **(a)** qRT-PCR validation of *GhAOX* transcript abundance in distinct transgenic overexpression lines, with *AtActin* serving as the internal reference gene. Data are presented as means ± SD from three independent experiments. Asterisks indicate statistically significant differences between overexpressed plants and wt controls (**P < 0.01; Student’s *t*-test). **(b)** Phenotypic observation of transgenic and WT seedlings. The growth rates of OE-GhAOX1a1/2–1 and OE-GhAOX2a1/2–2 were comparable to those of WT plants, while OE-GhAOX2b1/2–1 displayed accelerated seedling growth and OE-GhAOX2c1/2–2 exhibited significantly stunted growth. Histochemical staining revealed slightly reduced *in situ* accumulation of superoxide and H_2_O_2_ in *GhAOX*-overexpressing leaves relative to WT, as indicated by weakened NBT and DAB staining intensities (scale bar = 2 mm). **(c)** Quantitative comparison of leaf ROS staining. OE-GhAOX2a1/2–2 and OE-GhAOX2c1/2–2 showed milder NBT staining, whereas OE-GhAOX1a1/2–1 and OE-GhAOX2c1/2–2 exhibited markedly attenuated DAB staining compared with WT leaves (scale bar = 2 mm). **(d)** NBT staining of developing anthers at stages 6–10 showed no obvious differences in superoxide accumulation between transgenic lines and WT plants (scale bar = 200 μm). **(e)** DAB staining demonstrated significantly lower H_2_O_2_ levels in the anthers of OE-GhAOX2b1/2–1 and OE-GhAOX2c1/2–2 relative to WT during anther developmental stages 6-10 (scale bar = 200 μm).

To functionally validate the roles of *GhAOX* genes derived from Jin A in plant growth, microspore development, and ROS homeostasis, individual *GhAOX* members were heterologously overexpressed in *A. thaliana* via *Agrobacterium*-mediated genetic transformation. Multiple transgenic *Arabidopsis* lines with differential transcript abundances of exogenous *GhAOX* genes were successfully generated (**Figure 5a**). Phenotypic observation revealed that the overexpression of specific *GhAOX* isoforms significantly altered seedling growth performance (**Figure 5b**). Specifically, the growth rates of OE-*GhAOX*1a1/2–1 and OE-*GhAOX*2a1/2–2 transgenic lines were comparable to those of wt plants. In contrast, the OE-*GhAOX*2b1/2–1 line displayed accelerated seedling growth, while the OE-*GhAOX*2c1/2–2 line exhibited inhibited and stunted growth.

Given the well-established function of AOX proteins in ROS scavenging and redox homeostasis regulation, NBT and DAB histochemical staining were performed to quantify the *in situ* accumulation of superoxide anion and H_2_O_2_ in transgenic seedlings (**Figure 5b**). The weaker staining intensity in *GhAOX*-overexpressing lines indicated a significant reduction in endogenous ROS levels compared with wt seedlings, confirming the conserved ROS-scavenging capacity of cotton *GhAOX* proteins in planta.

ROS quantification and histochemical staining assays were further performed on rosette leaves of flowering transgenic plants (**Figure 5c**; Supplemental **Supplementary Table 4**). Consistent with the seedling-stage results, the accumulation levels of superoxide anion and H_2_O_2_ were significantly lower in OE-*GhAOX*2c1/2–2 transgenic plants relative to wt controls.

I_2_-KI pollen staining showed no obvious differences in pollen viability and stamen morphological development between all *GhAOX*-overexpressing transgenic lines and wild-type plants (Supplemental **Supplementary Figure 8**). NBT staining of anthers at developmental stages 6–10 revealed comparable superoxide anion accumulation between wild-type plants and four transgenic lines, including OE-*GhAOX*1a1/2-1, OE-*GhAOX*2a1/2-2, OE-*GhAOX*2b1/2-1, and OE-*GhAOX*2c1/2-2 (**Figure 5d**). In contrast, DAB staining demonstrated markedly reduced H_2_O_2_ accumulation in the anthers of OE-*GhAOX*2b1/2–1 and OE-*GhAOX*2c1/2–2 lines during stages 6-10 (**Figure 5e**). These results indicate that heterologous overexpression of *GhAOX2b1/2* and *GhAOX2c1/2* effectively alleviates H_2_O_2_ accumulation in developing anthers.

Furthermore, *in vitro* pollen germination assays were conducted to evaluate the effects of *GhAOX* overexpression on pollen development and growth (Supplemental **Supplementary Figure 9**; Supplemental **Supplementary Table 5**). No significant differences in pollen germination rate, pollen tube elongation rate, or pollen tube width were observed in OE-*GhAOX*2b1/2–1 and OE-*GhAOX*2c1/2–2 lines compared with wt plants. However, the OE-*GhAOX*1a1/2–1 line exhibited a distinctly decreased pollen germination rate, suggesting isoform-specific functional divergence among *GhAOX* family members.

**Figure 6:**
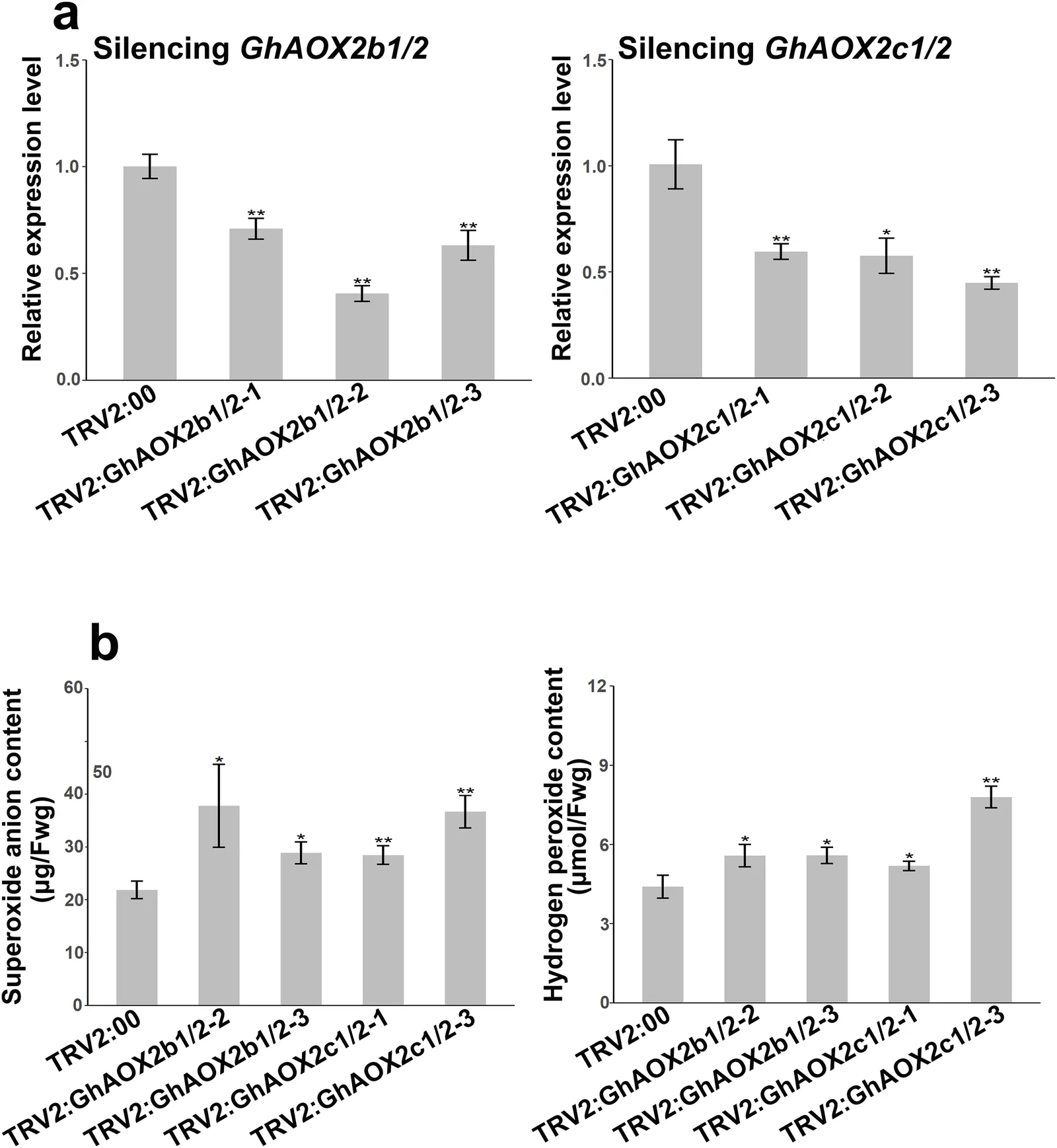
Functional validation of *GhAOX* gene silencing in the maintainer line Jin B. **(a)** qRT-PCR analysis of *GhAOX* transcript levels in *GhAOX*-silenced and empty-vector negative control plants (TRV2:00), with *GhEF1α* used as the internal reference gene. **(b)** Determination of superoxide anion and hydrogen peroxide contents in silenced and control plants. All data are presented as the means ± SD of three independent biological replicates. Asterisks indicate statistically significant differences between silenced lines and negative controls (*P < 0.05, **P < 0.01; Student’s *t*-test).

To further verify the ROS regulatory functions of *GhAOX2b1/2* and *GhAOX2c1/2* in cotton, VIGS was applied to specifically knock down the expression of these two genes in cotton seedlings. No visible phenotypic alterations were observed in VIGS-silenced plants compared with empty-vector control plants. qRT-PCR validation confirmed effective gene silencing: the transcript reduction efficiency of pTRV2:*GhAOX2b1/2* ranged from 40% to 70%, with an overall silencing efficiency of 30%-60%, while pTRV2:*GhAOX2c1/2* exhibited a transcript reduction rate of 45%-60% and a total silencing efficiency of 40%-55% (**Figure 6a**).

Subsequent ROS detection was performed on cotton leaves at 15 days post-VIGS infiltration (**Figure 6b**). Compared with the negative control (TRV2:00), both TRV2:*GhAOX2b1/2* and TRV2:*GhAOX2c1/2* silenced plants displayed significantly increased accumulation of superoxide anion and H_2_O_2_. These loss-of-function results further confirm that *GhAOX2b1/2* and *GhAOX2c1/2* serve as key regulators of ROS homeostasis in cotton, and their downregulation directly triggers excessive ROS accumulation in planta.

### 3.10 Screening of regulatory proteins interacting with *GhAOX2c1/2*

To further dissect the upstream regulatory mechanism governing *GhAOX2c1/2* transcriptional expression, a Y1H screening assay was performed using the *GhAOX2c1/2* promoter as the bait. A total of 32 positive clones associated with *GhAOX2c1/2* regulation were successfully identified. The homologous genes of these positive sequences were retrieved from *A. thaliana* and *Oryza sativa* via the UniProt database, followed by systematic annotation of their biological functions and subcellular localization characteristics. Among these candidate proteins, 10 were predicted to localize to the nucleus, including cell division control protein 2 homolog D, RGF1 INDUCIBLE TRANSCRIPTION FACTOR 1, ENHANCER OF AG-4 protein 2, Adagio protein 1, nuclear transport factor 2B, putative transcription elongation factor SPT5 homolog 1, nitrate regulatory gene 2 protein, one uncharacterized nuclear protein, and histone H2B (Supplemental **Supplementary Table 6**).

For pairwise Y1H validation, the coding sequences of these candidate prey proteins were individually inserted into the pGADT7 vector and transformed into *GhAOX2c1/2*-AbAi reporter yeast strains. Growth observations demonstrated that all validated prey proteins enabled stable growth of reporter strains on high-stringency selective medium, confirming authentic protein-promoter interactions between the candidate regulators and the *GhAOX2c1/2* promoter (Supplemental **Supplementary Figure 10**). Gene Ontology (GO) enrichment analysis indicated that these interacting proteins are functionally involved in multiple core biological processes, including cellular metabolism, biological regulation, and stimulus response (Supplemental **Supplementary Figure 11**).

Transcriptome-based expression profiling further revealed distinct expression patterns of these regulatory genes during the microspore abortion stage. Compared with Jin B, the expression level of *nitrate regulatory gene 2* was significantly upregulated in Jin A, whereas *histone H2B* expression was markedly downregulated (Supplemental **Supplementary Table 7**), implying their potential involvement in modulating *GhAOX2c1/2* expression during cotton anther abortion.

### 3.11 Differential accumulation and activity of *GhAOX* proteins during anther development

**Figure 7:**
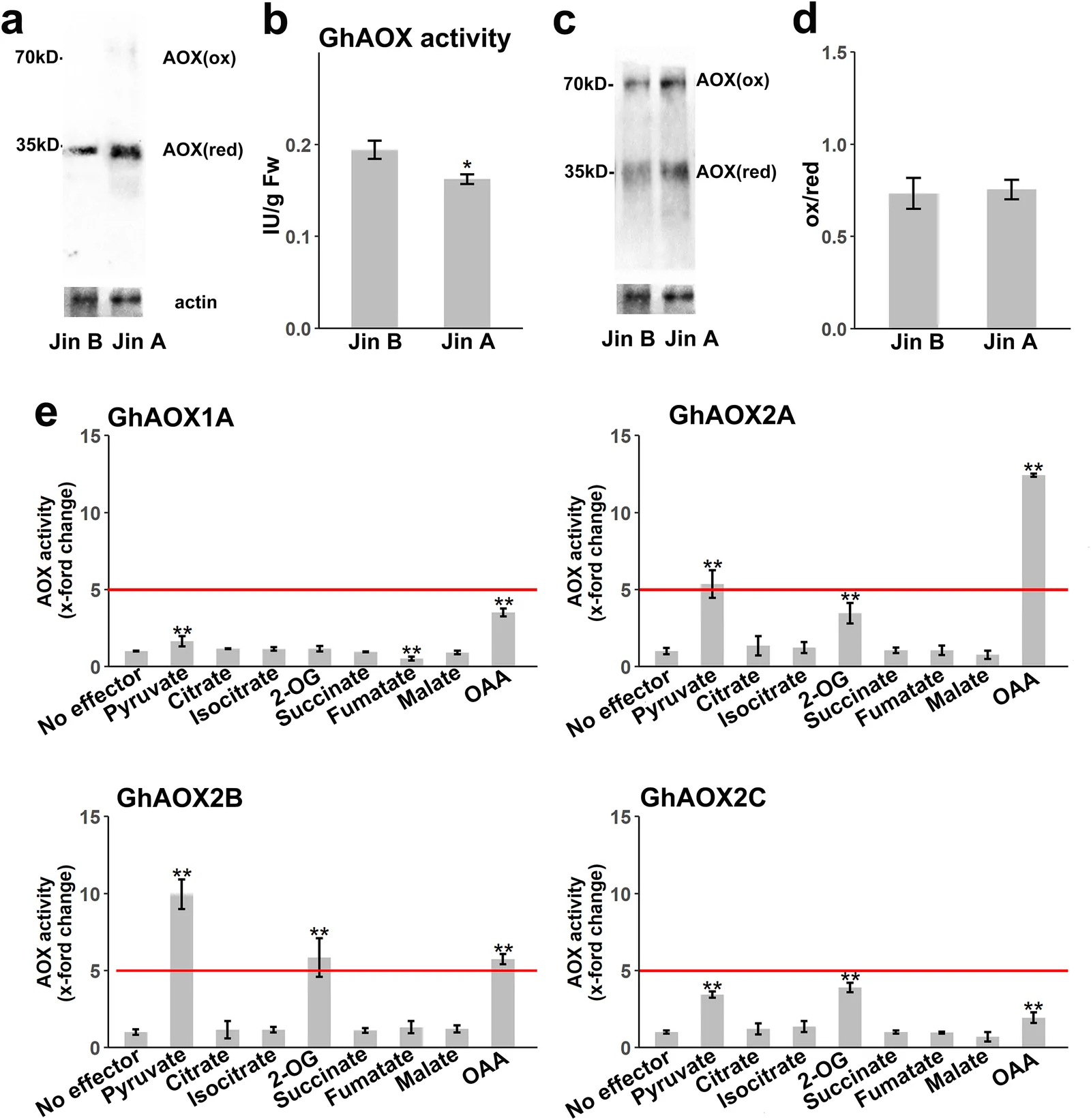
Accumulation, enzymatic activity, and post-translational regulation of *GhAOX* proteins in Jin A at the critical microspore abortion stage (Stage D). **(a)** Western blot quantification of total *GhAOX* protein using 13% SDS-PAGE with 0.1M DTT treatment, with cotton actin served as the protein loading control. **(b)** Determination of *GhAOX* enzymatic activity. **(c)** Western blot detection of *GhAOX* redox status using 13% native-PAGE without DTT treatment. **(d)** Statistical analysis of the *GhAOX* oxidized/reduced (ox/red) ratio. The grayscale values of protein bands were quantified using ImageJ software, with three independent experimental replicates. **(e)** Effects of different 2-oxo acid effectors on the enzymatic activities of *GhAOX1A1/2*, *GhAOX2A1/2, GhAOX2B1/2*, and *GhAOX2C1/2*. *GhAOX* activity was measured according to a previously described method (Li et al., 2012), using eight 2-oxo acids (5mM pyruvate, citrate, isocitrate, 2-OG, succinate, fumarate, malate, and OAA) as potential activators. *GhAOX* catalytic activity was determined by monitoring the absorbance variation derived from *GhAOX*-mediated oxidation of tetramethylhydroquinone. All enzyme activity data were normalized to the fold change of absorbance values obtained in the effector-free control group for intuitive comparison. All data are presented as the means ± SD of three independent biological replicates. Asterisks indicate significant differences between effector-treated and untreated groups (*P < 0.05, **P < 0.01; Student’s *t*-test). 2-OG, 2-oxoglutarate; OAA, oxaloacetate.

To clarify whether transcriptional alterations of *GhAOX* family members lead to variations in protein abundance during microspore abortion in Jin A, WB analysis was performed using denatured anther protein samples treated with DTT and SDS reducing agents (**Figure 7a**). WB results showed that the molecular weight of endogenous *GhAOX* proteins in both Jin A and Jin B was approximately 35 kDa, consistent with the theoretical size of reduced *GhAOX* isoforms. Using cotton actin as the internal reference, quantitative grayscale analysis revealed that the total *GhAOX* protein abundance was significantly higher in Jin A than in Jin B. Combined with tissue-specific expression profiles and protein quantification data, we speculated that *GhAOX*2B1/2 serves as the predominant functional isoform in Jin B, while *GhAOX*2C1/2 dominates *GhAOX* protein accumulation in Jin A anthers.

Notably, enzyme activity assays presented an opposite trend to protein abundance. During the critical microspore abortion stage, the total enzymatic activity of *GhAOX* in Jin A was significantly lower than that in Jin B (**Figure 7b**). These contradictory results indicate that the elevated *GhAOX* protein accumulation in Jin A cannot compensate for the impaired enzymatic activity, suggesting the occurrence of post-translational functional inhibition of *GhAOX* during cotton microspore abortion.

### 3.12 Redox status of *GhAOX* proteins and expression profiling of Trx/NTRC system

Post-translational redox modification acts as a crucial regulatory mechanism modulating AOX enzymatic activity. To explore whether redox state alteration contributes to the functional discrepancy between *GhAOX* protein abundance and activity in Jin A, non-reducing SDS-PAGE combined with WB was performed using protein samples prepared without DTT and SDS reducing agents (**Figure 7c**). Both monomeric (35 kDa) and dimeric (70 kDa) *GhAOX* protein forms were detected in the anthers of Jin A and Jin B, with higher total protein accumulation observed in Jin A. Quantitative grayscale analysis via ImageJ was further conducted to calculate the dimer/monomer ratio (oxidized/reduced form) of *GhAOX* proteins. The results showed that the dimer/monomer ratio of *GhAOX* in Jin B was 0.73, while no significant variation in this redox ratio was detected in Jin A (**Figure 7d**).

The Trx/NTRC system is the core regulatory module controlling plant AOX redox state transition. During microspore abortion, no significant expression differences of *Trx* and *NTRC* were observed between Jin A and Jin B (Supplemental **Supplementary Figure 12**).

### 3.13 Isoform-specific activation of *GhAOX* proteins by TCA cycle intermediates

Accumulating evidence has demonstrated that the enzymatic activation of plant AOXs strictly depends on specific 2-oxo acid or TCA cycle intermediates, and distinct AOX isoforms exhibit divergent responsiveness to different metabolic effectors. Given the isoform-specific sequence variations of *GhAOX* proteins in Jin A, we purified recombinant *GhAOX*1A1/2, *GhAOX*2A1/2, *GhAOX*2B1/2, and *GhAOX*2C1/2 proteins using a prokaryotic *E. coli* BL21 expression system. Subsequent effector activation assays were performed with seven typical TCA cycle intermediates to clarify the intrinsic mechanism underlying the mismatch between *GhAOX* protein abundance and enzymatic activity (**Figure 7e**).

Enzyme activity assays showed that three TCA intermediates, including pyruvate, OAA, and 2-OG, served as effective activators of *GhAOX* proteins, whereas citrate, isocitrate, succinate, and malate exerted negligible regulatory effects. Notably, distinct isoform-specific activation patterns were observed among *GhAOX* subfamily members. *GhAOX*1A1/2 was strongly activated by OAA and pyruvate, with a 2~3-fold increase in enzymatic activity, but was insensitive to 2-OG treatment; additionally, fumarate exhibited an inhibitory effect on *GhAOX*1A1/2 activity. In contrast, all three activators significantly enhanced the enzymatic activity of *GhAOX*2 subfamily members, with obvious functional divergence among isoforms. *GhAOX*2A1/2 and *GhAOX*2B1/2 displayed high sensitivity to TCA intermediates, with their activities increased by more than 5-fold upon effector induction. Specifically, OAA induced a greater than 9-fold activation of *GhAOX*2A1/2, and pyruvate triggered a over 9-fold activity enhancement of *GhAOX*2B1/2. By comparison, *GhAOX*2C1/2 exhibited markedly lower responsiveness, with the activation multiple of all three effectors less than 4-fold. Collectively, *GhAOX*2A1/2 and *GhAOX*2B1/2 were far more susceptible to TCA intermediate-mediated activation than *GhAOX*1A1/2 and *GhAOX*2C1/2.

To further verify the *in vivo* metabolic basis for differential *GhAOX* activation, the endogenous contents of pyruvate, OAA, and 2-OG in total bud cells and isolated mitochondria were quantified during the microspore abortion stage (**Table 2**). No significant differences in total and mitochondrial pyruvate contents were detected between Jin A and Jin B. The total 2-OG content was also comparable between the two lines; however, Jin A exhibited a significantly lower mitochondrial 2-OG accumulation than Jin B. Moreover, OAA contents at both the total cellular and mitochondrial levels were markedly reduced in Jin A relative to its maintainer line. The deficiency of mitochondrial 2-OG and OAA in Jin A at Stage D severely restricts the effector-dependent activation of *GhAOX* isoforms, ultimately leading to reduced overall *GhAOX* enzymatic activity despite elevated protein accumulation.

**Table 2:** Total and mitochondrial contents of pyruvate, 2-OG and OAA in Jin A and Jin B at Stage D.

Effector	Total		Mitochondria	
	Jin B	Jin A	Jin B	Jin A
Pyruvate	3.96 ± 0.23	3.82 ± 0.45	0.05 ± 0.01	0.05 ± 0.00
	μmol/g Fw	μmol/g Fw	μmol/mg pro	μmol/mg pro
2-OG	873.94 ± 56.77	739.11 ± 83.43	132.34 ± 5.70	101.13 ± 7.89**
	nmol/g Fw	nmol/g Fw	nmol/mg pro	nmol/mg pro
OAA	215.74 ± 14.16	182.34 ± 6.74*	3.87 ± 0.07	3.33 ± 0.25*
	μg/g Fw	μg/g Fw	μg/mg pro	μg/mg pro

All data are presented as the means ± SD of three independent biological replicates. Asterisks represent statistically significant differences between Jin A and Jin B (* *P* < 0.05, ** *P* < 0.01; Student’s *t*-test). 2-OG, 2-oxoglutarate; OAA, oxaloacetate.

## 4 Discussion

### 4.1 Tissue specificity and sensitivity to stress of *GhAOX* members

Consistent with most plants, including *Pisum sativum*, rapeseed, moso bamboo, and *Aquilaria sinensis*, the *GhAOX* gene family was phylogenetically classified into two distinct subfamilies, *GhAOX1* and *GhAOX2* (Yang et al., 2019; Liu et al., 2020; Wang et al., 2022; Rodrigues et al., 2023). Similar to the evolutionary characteristics of other cotton gene families, segmental duplication served as the predominant driving force for *GhAOX* family expansion (Chen et al., 2022). Notably, plant *AOX* lineages exhibited species-specific evolutionary divergence. In *A. thaliana*, wheat, and *Commiphora myrrha*, *AOX* family expansion was mainly confined to the *AOX1* subfamily (Polidoros et al., 2009; Liu et al., 2020; Zhang et al., 2023b). In contrast, prominent expansion of the *AOX2* subfamily had been observed in cotton, rapeseed, and soybean (Yang et al., 2019; Sweetman et al., 2022). Such lineage-specific evolutionary patterns contributed to the functional diversification of AOX paralogs and enabled plants to achieve precise and flexible adaptive responses to variable environmental conditions. Consistent with the conserved function of AOXs across plant species, overexpression and VIGS-mediated silencing assays in this study verified that *GhAOX* proteins possess canonical ROS-scavenging activity. Previous studies had demonstrated that AOX overexpression generally promotes plant growth and development (Giraud et al., 2008). However, the present study observed an opposite phenotypic change, in which heterologous overexpression of*GhAOX2b1/2* inhibited seedling growth in *Arabidopsis*. This discrepancy indicated that plant growth and stress tolerance relied on the precise spatiotemporal modulation of AOX expression, which balanced the dual functional roles of AOX in developmental regulation and stress defense.

Subfamily-specific spatial expression and divergent stress responsiveness further reflected the functional specialization of plant AOX paralogs. In *Arabidopsis* and tomato, *AOX1* subfamily genes were predominantly expressed in floral tissues, vegetative organs, and roots, with particularly high abundance in stamens (Saisho et al., 1997; Simons et al., 1999; Considine et al., 2002). This expression pattern was highly consistent with our findings in cotton, in which *GhAOX* genes exhibited preferential expression in floral organs, especially stamens, compared with vegetative roots, stems, and leaves.

Accumulated evidence had confirmed that plant *AOX1* genes were broadly responsive to diverse biotic and abiotic stresses, as well as mitochondrial respiratory signals (Considine et al., 2002). In *Arabidopsis*, *AtAOX1a* was significantly induced by pathogen infection, proline, and alanine treatments, whereas *AtAOX1b* and *AtAOX1c* remained transcriptionally stable under the same conditions (Considine et al., 2002; Oh et al., 2022, 2023). Similarly, sweet potato *AOX1a* and *AOX1b* were rapidly upregulated in response to low-temperature stress (Zhang et al., 2021). By comparison, *AOX2* subfamily genes were generally insensitive to mitochondrial inhibitors and low-temperature stimuli in *Arabidopsis*, soybean, and sweet potato (Saisho et al., 1997, 2001; Considine et al., 2002; Zhang et al., 2021). Nevertheless, the present study identified a distinct regulatory pattern of cotton *GhAOX* genes. In upland cotton, *GhAOX2* subfamily members, particularly *GhAOX2c1/2*, displayed dramatic transcriptional changes in response to osmotic stress (PEG), salt stress (NaCl), cold stress, and heat stress. This stress-responsive pattern differed from that in *Arabidopsis* and sweet potato but was consistent with observations in legume species such as chickpea, in which *AOX2d* was strongly induced by cold, drought, and salt stresses (Costa et al., 2014; Sweetman et al., 2019, 2020). Additionally, low temperature specifically activated *AOX2a* expression in carrot without significantly affecting *AOX1* transcription (Campos et al., 2021), further supporting the unique stress-adaptive function of *AOX2* paralogs in specific plant lineages.

Although *GhAOX1* had been reported to respond to multiple exogenous stimuli, including low temperature, citric acid, salicylic acid, and mitochondrial inhibitors (Li et al., 2008), our temporal expression profiling revealed fine-scale differential responses of *GhAOX* paralogs. *GhAOX1a1/2* exhibited transient early induction, being significantly upregulated within 1 h of salt and heat stress and during the initial stage of cold stress, indicative of its specific role in early stress signal perception. As stress duration increased, *GhAOX1a1/2* expression gradually declined, while *GhAOX2b1/2* and *GhAOX2c1/2* maintained sustained high expression, suggesting their functional specialization in long-term stress adaptation. Notably, *GhAOX2a1/2* maintained consistently low transcript levels across all tissues and stress conditions, implying a limited functional contribution to cotton growth, development, and stress tolerance.

The subfamily-specific and tissue-preferential expression patterns of *GhAOX* genes were primarily determined by differential cis-regulatory element compositions in their promoter regions. The relatively low *GhAOX* expression in roots might be attributed to the lack of light signal activation, which restricted the function of light-responsive cis-elements under soil-growth conditions. Promoter analysis further demonstrated that *GhAOX2b1/2* and *GhAOX2c1/2* were enriched with cis-elements associated with phytohormone signaling and flavonoid biosynthesis. Floral organs, especially anthers, possessed active hormone signaling and abundant flavonoid accumulation, which might specifically activate the transcription of *GhAOX2b1/2* and *GhAOX2c1/2*, thereby driving their anther-preferential expression. In terms of stress responsiveness, the high abundance of abscisic acid- and ethylene-related cis-elements in the promoters of *GhAOX2b1/2* and *GhAOX2c1/2* provided a core molecular basis for their robust stress-induced transcription. The mechanistic link between promoter composition and *AOX* transcriptional regulation represents a key focus for future investigation. Elucidating the regulatory networks governing *AOX* expression in cotton will provide deeper insights into the molecular mechanisms underlying developmental plasticity and stress adaptation in this globally important crop.

### 4.2 The key that determined *GhAOX* activity in Jin A

In this study, we further revealed that *GhAOX* function in Jin A was modulated by a sophisticated multimodal regulatory network consisting of transcriptional reprogramming, protein redox modulation, and TCA-derived metabolic activation. At the transcriptional level, MRR served as a core module shaping the isoform-specific expression pattern of *GhAOX*. In *Arabidopsis*, multiple MRR-related transcription factors had been identified to positively drive *AtAOX1a* expression (Giraud et al., 2009; Ng et al., 2013; Van Aken et al., 2013; Zhang et al., 2017). A previous study in CMS *Brassica juncea* demonstrated that General Regulator 3 modulates *AOX1a* transcription and interacts with the AOX repressor MYB29 to coordinate glucosinolate metabolism (Li et al., 2022). However, the MRR cascade governing CMS-associated pollen abortion remained poorly characterized. Here, we screened a set of candidate regulatory proteins that directly bind to the *GhAOX2c1/2* promoter, and GO functional annotation confirmed their essential roles in transcriptional regulation. Notably, we detected differential expression of *Histone H2B* between Jin A and Jin B. As a core chromatin structural protein, Histone H2B participated in multiple fundamental biological processes, including transcriptional modulation, DNA repair, replication, and chromosomal stabilization (Gaudet et al., 2011). The downregulation of *Histone H2B* in Jin A might induce nucleosome relaxation and increase the chromatin accessibility of downstream target loci, thereby facilitating transcriptional reprogramming. Accumulating evidence had proven that histone modification acts as a pivotal epigenetic mechanism to fine-tune anther development and plant growth via dynamic regulation of gene transcription (Weake and Workman, 2008; Cao et al., 2015). Consistent with this notion, our unpublished data further revealed elevated ubiquitination levels and increased acetylation modification sites of Histone H2B in Jin A. Collectively, these findings indicate that altered expression and post-translational modification of Histone H2B might function as a key epigenetic switch to govern the transcriptional abundance of *GhAOX2c1/2* during cotton CMS abortion.

Beyond transcriptional control, the catalytic activity of *GhAOX* was dynamically modulated by protein redox status. The oxidized *GhAOX* homodimer, formed via intermolecular disulfide crosslinking at the conserved CysI residue, represented the catalytically inactive storage form, whereas thiol-dependent reduction disrupted disulfide bonding to generate non-covalent, enzymatically active *GhAOX* monomers. Previous studies in maize and soybean had verified that alternative respiratory flux was strictly constrained by the sulfhydryl/disulfide redox balance of AOX proteins (Umbach and Siedow, 1993; Karpova et al., 2002). In this study, no significant transcriptional differences in *Trx* and *NTRC* were detected between Jin A and Jin B during microspore abortion. Furthermore, the monomer/dimer ratio of *GhAOX* proteins remained comparable in the two lines. These results demonstrated that redox-state modification of *GhAOX* proteins contributed minimally to the reduced AOX enzymatic activity in the Jin A CMS line.

Metabolite-dependent allosteric activation by TCA cycle intermediates constituted the dominant regulatory mode of *GhAOX* enzymatic function. It was well established that plant AOX activity was strictly dependent on allosteric stimulation by specific carboxylate metabolites (Millar et al., 1993; Umbach et al., 2006; Selinski et al., 2017). The 2-oxo acid-mediated activation of AOX was highly sophisticated, relying on the combined effects of three conserved cysteine residues (CysI, CysII, and CysIII) and the chemical properties of specific effector molecules (Umbach and Siedow, 1996, 2002; Selinski et al., 2017). Early site-directed mutagenesis studies confirmed that CysI acts as the core functional site for both disulfide bond formation and 2-oxo acid-dependent activation (Rhoads et al., 1998; Vanlerberghe et al., 1998). In *Arabidopsis* and soybean, distinct AOX isoforms were selectively activated by pyruvate, glyoxylate, 2-OG, or OAA (Day and Wiskich, 1995; Millar et al., 1996; Selinski et al., 2018a). Comparative sequence analysis revealed universal conservation of the CysI residue across all functional AOX homologs, while natural substitutions at CysI in specific species (including tomato AOX1B, lotus AOX1A/1B, rice AOX1B, maize AOX3, and *Trypanosoma brucei* AOX) drastically altered effector specificity or conferred constitutive enzyme activity independent of metabolite activation (Ito et al., 1997; Djajanegara et al., 1999; Karpova et al., 2002; Holtzapffel et al., 2003; Grant et al., 2009; Selinski et al., 2017, 2018a; Xu et al., 2021). In contrast, the functional role of CysII remained ambiguous, with multiple studies reporting that CysII substitutions exerted negligible effects on effector-induced AOX activation (Umbach et al., 2002; Selinski et al., 2017; Sankar et al., 2022). CysIII exhibited high evolutionary polymorphism across plant lineages, and leucine substitution at the CysIII site in *Arabidopsis* AtAOX1C/1D generated hyperactive isoforms with effector-independent constitutive activity (Selinski et al., 2017). In cotton, CysI was strictly conserved among all *GhAOX* isoforms, indicating conserved evolutionary mechanisms underlying 2-oxo acid-mediated activation. All four *GhAOX* isoforms can be activated by pyruvate, 2-OG, and OAA, yet with striking differences in activation efficiency. Notably, serine substitution at the CysII site of *GhAOX*2A1/2 did not eliminate its sensitivity to pyruvate. By contrast, natural leucine substitution at the CysIII site of *GhAOX*2A1/2 and *GhAOX*2C1/2 potentially enhanced their basal activation capacity relative to *GhAOX*1A1/2, consistent with previously reported isoform-specific functional divergence.

Isoform-specific differential metabolite responsiveness was a conserved functional feature of plant AOX families. In *Arabidopsis*, AtAOX1A was robustly activated by pyruvate, 2-OG, and OAA, whereas AtAOX1C was completely insensitive to these effectors and AtAOX1D was only activated by 2-OG (Selinski et al., 2018a). Legume AOX2 isoforms also exhibited divergent pyruvate sensitivity: root-specific AOX2D and cotyledonary AOX2A/AOX2D displayed a nearly 10-fold difference in pyruvate activation K1/2 values, and GmAOX2D showed stronger activation potential than GmAOX2A under saturated pyruvate conditions (Finnegan et al., 1997; Selinski et al., 2018b; Sweetman et al., 2022). Consistent with these findings, our results revealed prominent functional divergence among cotton *GhAOX* isoforms. *GhAOX*1A1/2 was barely activated by 2-OG. Most notably, although *GhAOX*2C1/2 could be induced by pyruvate and OAA, its activation fold (< 5-fold) was markedly lower than that of *GhAOX*2A1/2 and *GhAOX*2B1/2. Such differential activation patterns were presumably attributed to subtle structural variations among isoforms. Further site-directed mutagenesis and structural biology analyses were required to identify the key amino acid residues and conserved motifs determining *GhAOX* isoform-specific activity. In addition, quantitative metabolic detection revealed significant depletion of mitochondrial 2-OG and OAA in Jin A during microspore abortion. This *in vivo* deficiency of core activating metabolites acted synergistically with the low intrinsic effector sensitivity of *GhAOX*2C1/2, ultimately leading to impaired overall *GhAOX* catalytic activity in the CMS line.

In summary, this multi-layered regulatory model fully explained the uncoupling phenomenon between *GhAOX* protein abundance and enzymatic activity in Jin A. During the critical microspore abortion stage, perturbed *GhAOX* transcription remodeled the isoform composition of *GhAOX* proteins: *GhAOX2c1/2* was significantly upregulated, while *GhAOX2b1/2* was suppressed, resulting in *GhAOX*2C1/2 becoming the dominant isoform in abortive anthers. Although the Trx/NTRC-mediated redox status of total *GhAOX* proteins remained unchanged between Jin A and Jin B, *GhAOX*2C1/2 possessed intrinsically low responsiveness to core mitochondrial activators (2-OG and OAA). Furthermore, *in vivo* depletion of mitochondrial 2-OG and OAA in Jin A further restricted metabolite-dependent *GhAOX* activation. Collectively, the epigenetic and transcriptional reprogramming of *GhAOX*, isoform-specific functional divergence, and mitochondrial metabolic deficiency jointly triggered the decoupling of *GhAOX* protein accumulation and catalytic activity. This multilayered dysregulation disrupted mitochondrial ROS homeostasis, causes excessive ROS burst, and ultimately induces tapetal abnormality and pollen abortion in cotton CMS line Jin A.

### 4.3 Relationship between *GhAOX* and CMS

Mitochondrial respiratory complex dysfunction acted as a core upstream trigger for AOX transcriptional activation, establishing a tight regulatory linkage between AOX modulation and CMS progression. Perturbations in mitochondrial complex composition and functionality were well-documented hallmarks of plant CMS, providing a molecular bridge connecting AOX-mediated respiratory homeostasis and pollen fertility regulation. In maize, Complex I mutations (*NCS2*) substantially induced *AOX2* expression, recapitulating the respiratory perturbation triggered by rotenone inhibition. Similarly, reduced accumulation of COX2 in *NCS6* mutants elevated *AOX3* transcription, consistent with the AOX-inductive effects of cyanide-mediated COX pathway inhibition (Newton et al., 2004). In cotton, recent research had further validated this regulatory association: the chimeric ATP synthase gene *orf610a*, a key CMS-associated factor, significantly upregulates *GhAOX* expression, with a particularly pronounced effect on *GhAOX2c1/2* (Han et al., 2025). Combined with our findings regarding *GhAOX* dysregulation in Jin A, these lines of evidence solidified the conserved correlation between mitochondrial complex dysfunction, AOX transcriptional reprogramming, and CMS occurrence. Our previous study confirmed that excessive superoxide anion accumulation in Jin A primarily originated from electron leakage at mitochondrial Complex III (Zhang et al., 2023a). Nevertheless, whether the specific upregulation of *GhAOX2c1/2* is directly triggered by Complex III dysfunction remains to be experimentally validated. Notably, a recent mammalian study demonstrated that AOX expression prolonged the survival of mice carrying Complex III mutations (Jacobs et al., 2023), providing a valuable reference for dissecting the functional interaction between AOX and Complex III in plant CMS systems.

ROS homeostasis perturbation represented another pivotal mechanistic link between *GhAOX* dysregulation and cotton pollen abortion. Compelling evidence had established that aberrant mitochondrial ROS overaccumulation served as a universal initiating signal for microspore abortion in multiple plant CMS systems (Zhou et al., 2019; Ma et al., 2022; Zhang et al., 2023a). As a canonical mitochondrial protective protein, AOX alleviated respiratory electron leakage and restricts excess ROS generation. Importantly, ROS burst acted as a critical mitochondrial retrograde signal to activate AOX transcription under conditions of mitochondrial dysfunction and environmental stress (Murik et al., 2019; He et al., 2019, 2021; Fedotova et al., 2023; Manbir et al., 2022; Aziz et al., 2022; Qiao et al., 2023; Analin et al., 2024). In the Jin A CMS line, Complex III-derived ROS accumulation strongly induced *GhAOX2c1/2* transcription, which presumably functions as a compensatory feedback mechanism to constrain oxidative damage during anther development. However, this transcriptional induction failed to achieve functional compensation, as elevated *GhAOX* protein abundance was not accompanied by enhanced catalytic activity. Further mechanistic dissection confirmed that multi-layered regulatory defects, including isoform-specific transcriptional reprogramming, intrinsically low metabolite sensitivity of dominant *GhAOX* isoforms, and *in vivo* deficiency of TCA-derived activators, collectively induce severe uncoupling between *GhAOX* protein accumulation and enzymatic function. This regulatory mismatch disrupted mitochondrial redox equilibrium, triggers sustained ROS overburst, and ultimately mediated tapetal programmed cell death and pollen abortion in Jin A.

The regulatory characteristics and functional variability of AOX also provided novel insights for cotton hybrid breeding and germplasm improvement. The concept of AOX-assisted crop selection had recently emerged as a promising strategy to enhance plant stress resilience and broad-spectrum adaptability (Arnholdt-Schmitt et al., 2024). AOX-mediated respiratory homeostasis was regarded as a reliable biochemical indicator of plant vigor, with the potential to mitigate the adverse cytoplasmic effects associated with CMS. In cotton hybrid breeding, the development of high-quality fertile restorer lines remained a core research priority (Li et al., 2023). Sterile cytoplasmic backgrounds commonly exert pleiotropic detrimental effects on cotton agronomic performance, including defective anther development, abnormal organ morphogenesis, reduced yield potential, and impaired fiber quality (Li et al., 2023). At the cellular level, CMS cytoplasm induced chronic mitochondrial ROS accumulation, which further suppressed oxidative phosphorylation and exacerbated mitochondrial functional defects. As a pivotal ROS-detoxifying module, enhanced AOX capacity and activity could effectively sustain mitochondrial electron flux, alleviate oxidative damage, and strengthen plant stress adaptability. Moreover, specific AOX isoforms positively regulated vegetative growth and biomass accumulation. Therefore, utilizing AOX activity and regulatory status as molecular and biochemical markers represented a feasible and high-efficiency strategy for screening elite restorer lines and optimizing cotton hybrid breeding systems.

Nevertheless, the functional complexity and isoform divergence of the *GhAOX* family imposed critical constraints on its breeding application. Individual *GhAOX* paralogs exhibited distinct, even opposing, biological functions in regulating plant growth and development. Our heterologous expression assays verified that *GhAOX2b1/2* overexpression promoted seedling growth, whereas ectopic overexpression of *GhAOX2c1/2* inhibited early seedling development in *Arabidopsis*. These divergent phenotypic effects indicated that simple overexpression of a single *GhAOX* isoform could not effectively offset the adverse cytoplasmic effects of CMS. Instead, coordinated expression of multiple *GhAOX* members, balanced *GhAOX* redox status, and stable mitochondrial metabolic microenvironment were essential to maintain normal mitochondrial function and reproductive development in hybrid progenies. Accordingly, our future research will focus on two key directions: (1) systematic screening and functional characterization of elite cotton restorer lines; and (2) validating the potential of AOX catalytic activity and regulatory patterns as high-throughput molecular markers for fertile hybrid progeny identification and superior restorer line breeding.

## 5 Conclusion

In summary, a total of eight *GhAOX* family members were systematically identified in upland cotton. Among them, *GhAOX2b1/2* and *GhAOX2c1/2* exhibited predominant expression in stamens and displayed broad transcriptional responsiveness to multiple abiotic stresses, including PEG-simulated osmotic stress, salt stress, cold stress (4°C), and heat stress (37°C). Importantly, this study uncovered a prominent functional decoupling between *GhAOX* protein abundance and enzymatic activity during microspore abortion in the cotton CMS line Jin A. This inconsistent phenomenon was synergistically determined by three key factors: isoform-specific differential expression of *GhAOX* family genes, the intrinsically low sensitivity of *GhAOX*2C1/2 to TCA-cycle activator effectors, and the significant deficiency of mitochondrial oxaloacetate and 2-oxoglutarate in abortive anthers. These findings provide a novel mechanistic interpretation for mitochondrial ROS overaccumulation during pollen abortion in CMS cotton and deepen our mechanistic understanding of how altered intracellular microenvironments reshape *GhAOX* enzymatic activity and molecular functions in plant reproductive development.

## 6 Accession numbers

Sequence data for the genes from this article can be found in the Uniprot, NCBI or CottonFGD databases under the following numbers: *Ghir_A12G028850* (*GhAOX1a1*), *Ghir_D12G029000* (*GhAOX1a2*), *Ghir_A03G019170* (*GhAOX2a1*), *Ghir_D02G020510* (*GhAOX2a2*), *Ghir_A03G019180* (*GhAOX2b1*), *Ghir_D02G020520* (*GhAOX2b2*), *Ghir_A04G012310* (*GhAOX2c1*), *Ghir_D04G016730* (*GhAOX2c2*), *Ghir_A05G017800* (*GhAOX4a1*), *Ghir_D05G017790* (*GhAOX4a2*), AT3G22370 (AtAOX1A), AT3G22360 (AtAOX1B) AT3G27620 (AOX1C), AT1G32350 (AOX1D), AT5G64210 (AtAOX2), Q9M432 (white poplar aox1b), Q9SC31 (white poplar aox1), Q07185 (soybean AOX1), O03376 (soybean AOX3), Q41266 (soybean AOX2), A0A061E955 (*Theobroma cacao* AOX2), A0A061G6E7 (*Theobroma cacao* AOX1), A0A061GHF5 (*Theobroma cacao* AOX4), Q40294 (mango AOMI1), Q41224 (tobacco AOX1), Q40578 (tobacco AOX2), O82807 (rice AOX1A), Q8W855 (rice AOX1C), O82766 (rice AOX1B), A0A3L6EQ26 (maize AOX1A_0), A0A3L6FWX8 (maize AOX4_0), A0A3L6FX45 (maize AOX4_1), A0A3L6G163 (maize AOX1A_2), A0A8J8Y6L7 (maize AOX1B), Q26710 (*Trypanosoma brucei* brucei AOX), C0SUJ4 *(Nelumbo nucifera* AOX1A), C0SUJ5 (*Nelumbo nucifera* AOX1B), Q84V46 (*Solanum lycopersicum* AOX1B).

## Data Availability

The original contributions presented in the study are included in the article/**Supplementary Material**. Further inquiries can be directed to the corresponding authors.
