# Supplementary material for: Genome-wide identification of GhAOX gene family and its expression and regulation in cotton CMS line Jin A
Source: Front Plant Sci. 2026 Aug 14;17:1898123. doi: 10.3389/fpls.2026.1898123 (PMC13523418; doi:10.3389/fpls.2026.1898123)
Supplement: Supplementary file 1 [file DataSheet2.pdf]

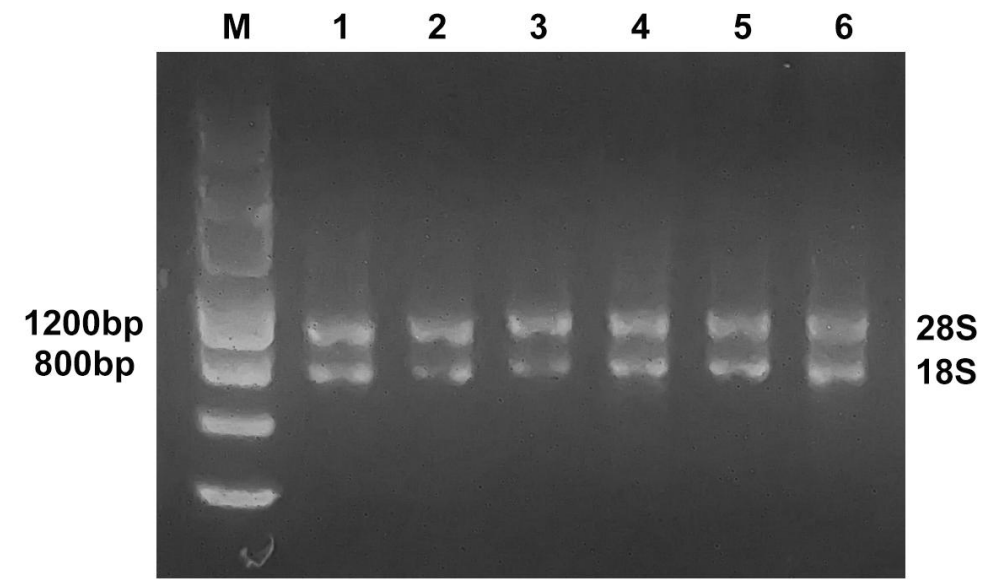

Supplemental Figure S1.

Agarose gel electrophoresis of total RNA extracted from Jin A and Jin B at Stages B, D, and A. RNA integrity was verified using 1% agarose gel electrophoresis, which revealed distinct, sharp bands corresponding to 28S and 18S rRNA. The 5S rRNA band was faint and barely detectable. The 28S rRNA band exhibited greater band intensity and width relative to the 18S rRNA band, with no smearing or trailing observed across all samples. These results confirmed that the extracted total RNA was of high integrity and quality. Channels 1 to 6 corresponded to Jin B at Stage B, Jin A at Stage B, Jin B at Stage D, Jin A at Stage D, Jin B at Stage A, and Jin A at Stage A, respectively.

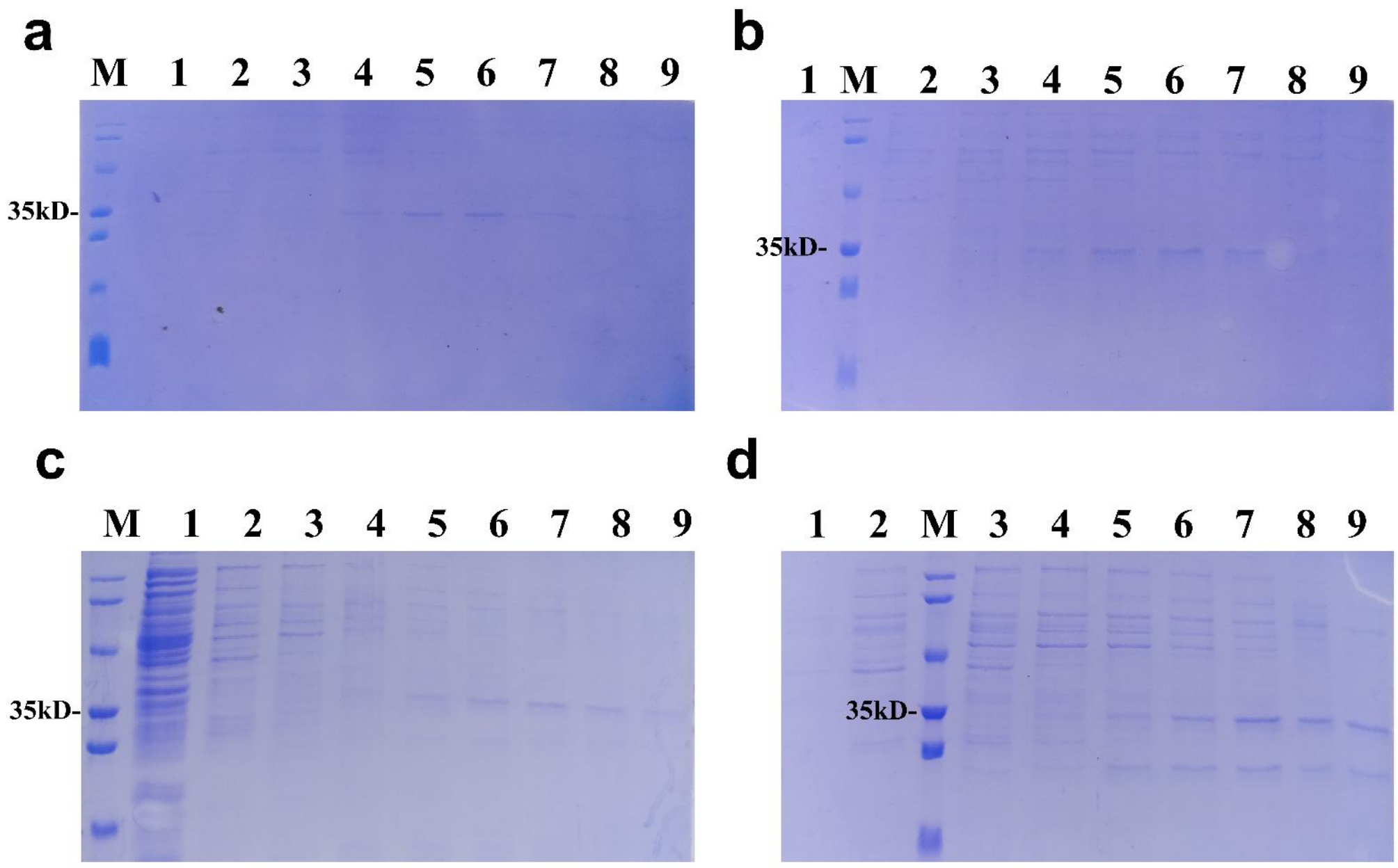

Supplemental Figure S2.

SDS-PAGE verification of purified recombinant GhAOX proteins. The purified isoforms were successfully detected in the corresponding gel lanes: **(a)** *GhAOX1A1/2* in lanes 5 and 6, **(b)** *GhAOX2A1/2* in lanes 5, 6, and 7, **(c)** *GhAOX2B1/2* in lanes 6, 7, and 8, and **(d)** *GhAOX2C1/2* in lanes 7, 8, and 9.

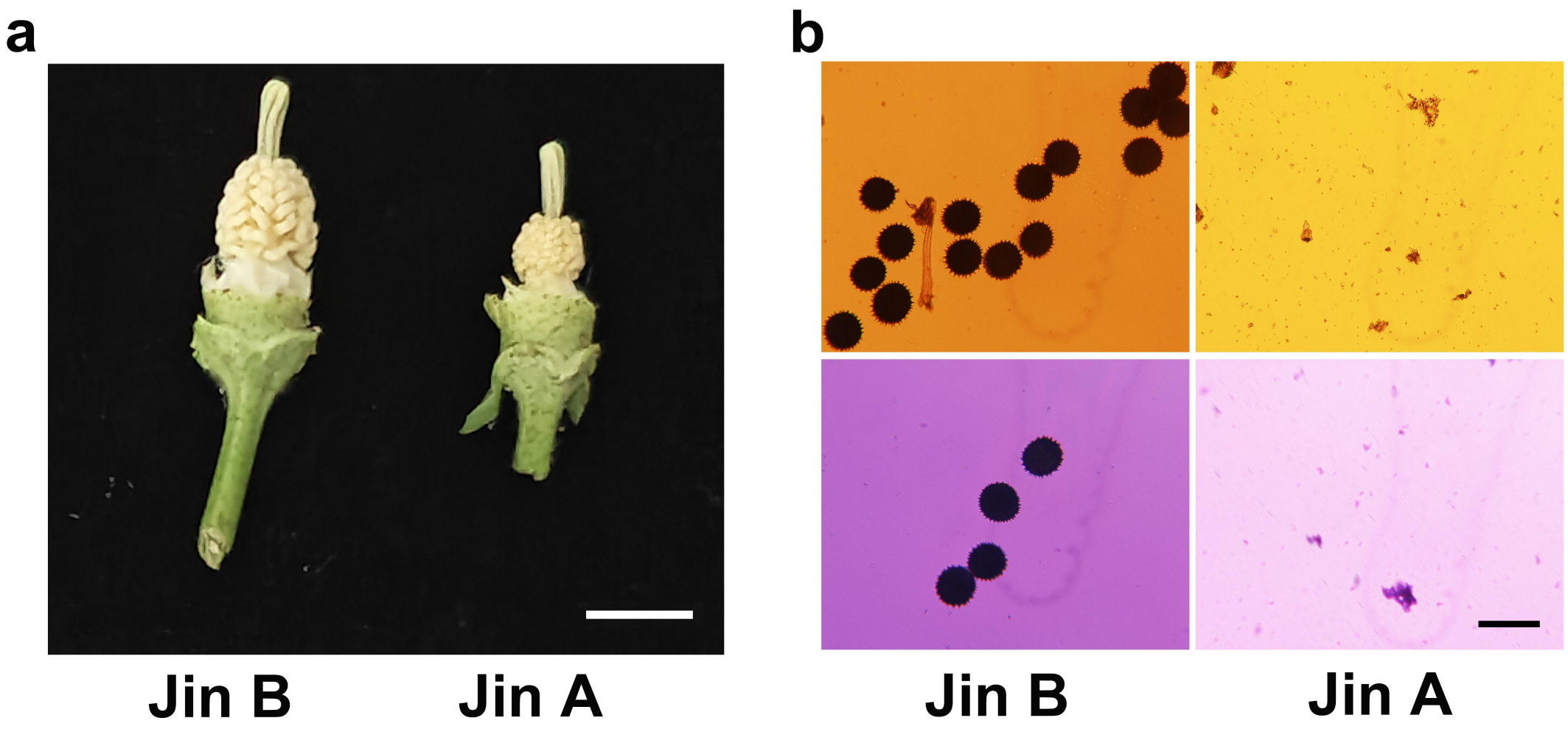

Supplemental Figure S3.

Phenotypic characterization of anthers and pollen grains from the CMS line Jin A and its maintainer line Jin B. **(a)** Morphological comparison of mature anthers from Jin A and Jin B (scale bar = 10 mm). **(b)** Pollen viability assessment via I_2-KI staining (upper panel) and Alexander staining (lower panel) in Jin A and Jin B (scale bar = 100 μm).

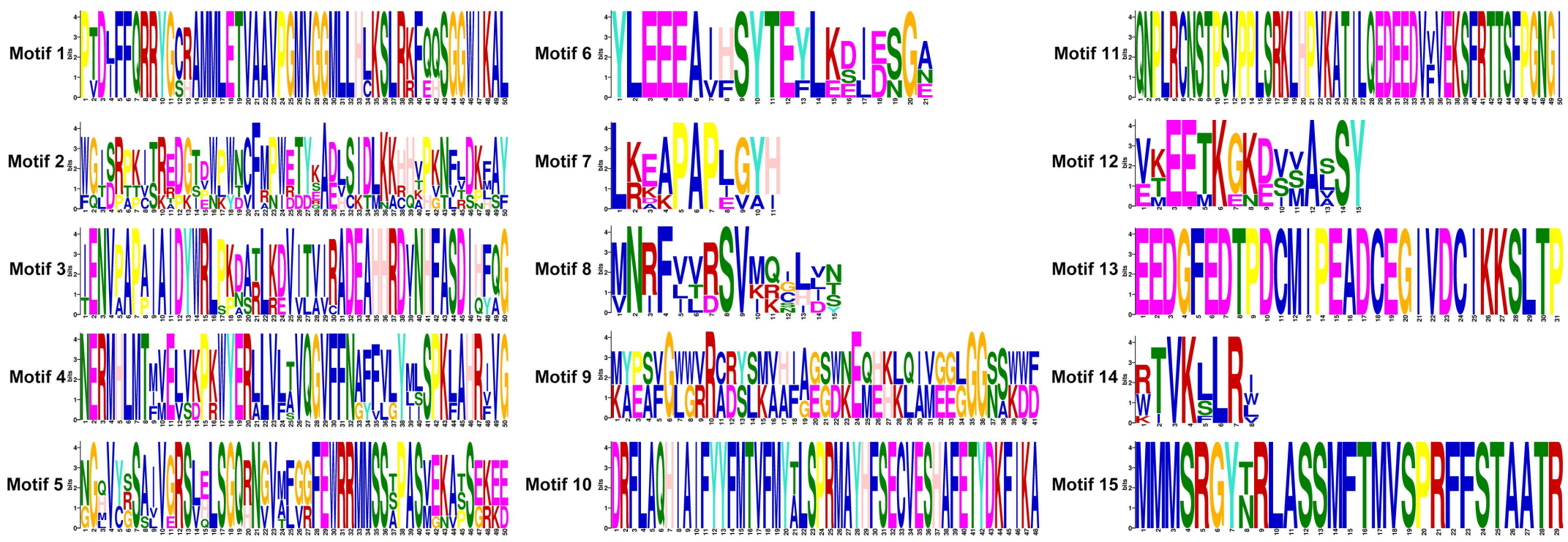

Supplemental Figure S4.

Basic information regarding conserved motifs of cotton AOX family proteins. Conserved motifs within GhAOX protein sequences were identified using the Multiple Em for Motif Elicitation (MEME) tool.

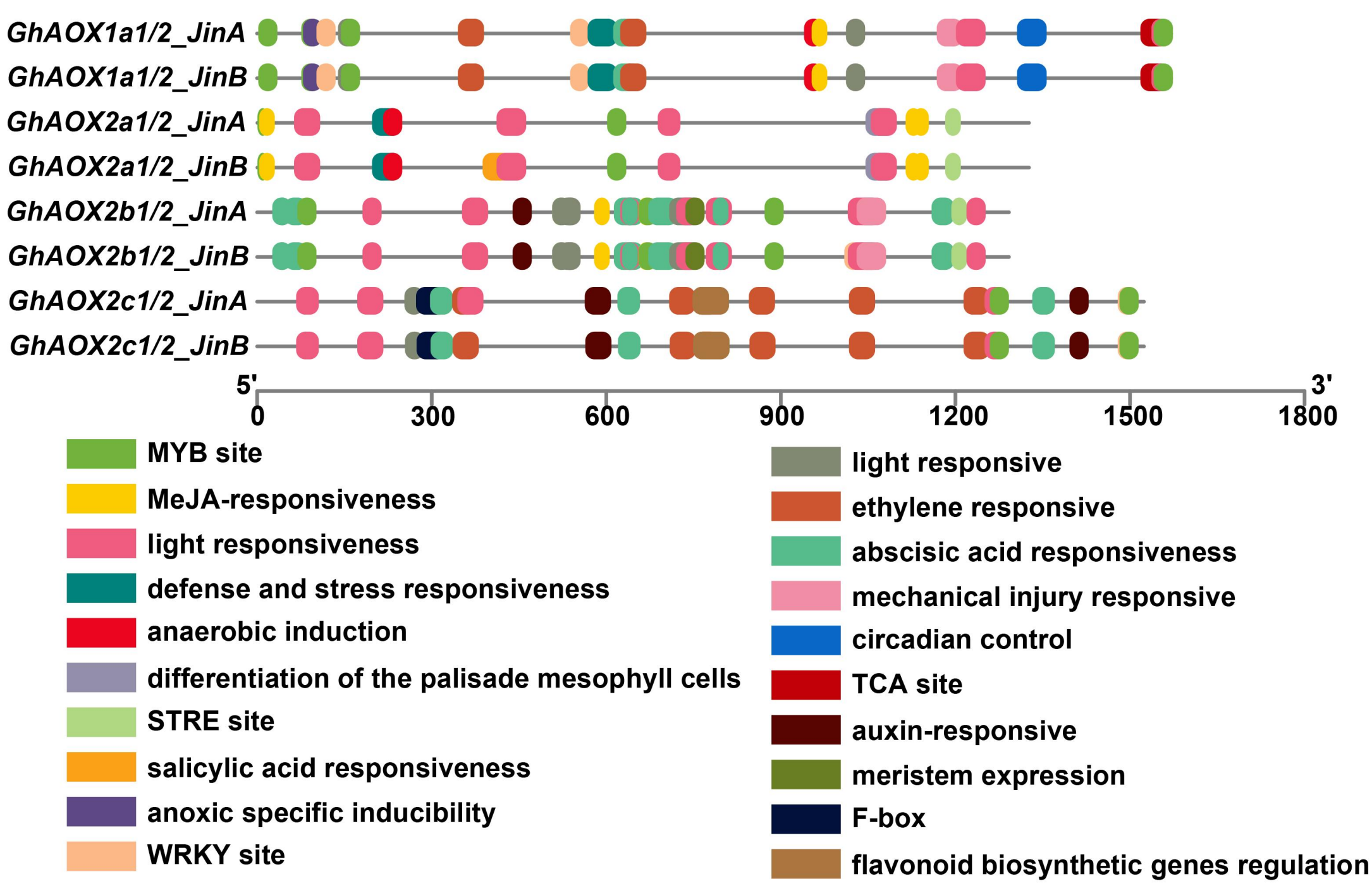

Supplemental Figure S5.

Analysis of cis-acting regulatory elements in the promoter regions of *GhAOX* family genes. A total of 175 putative cis-acting elements were identified across all *GhAOX* promoter sequences.

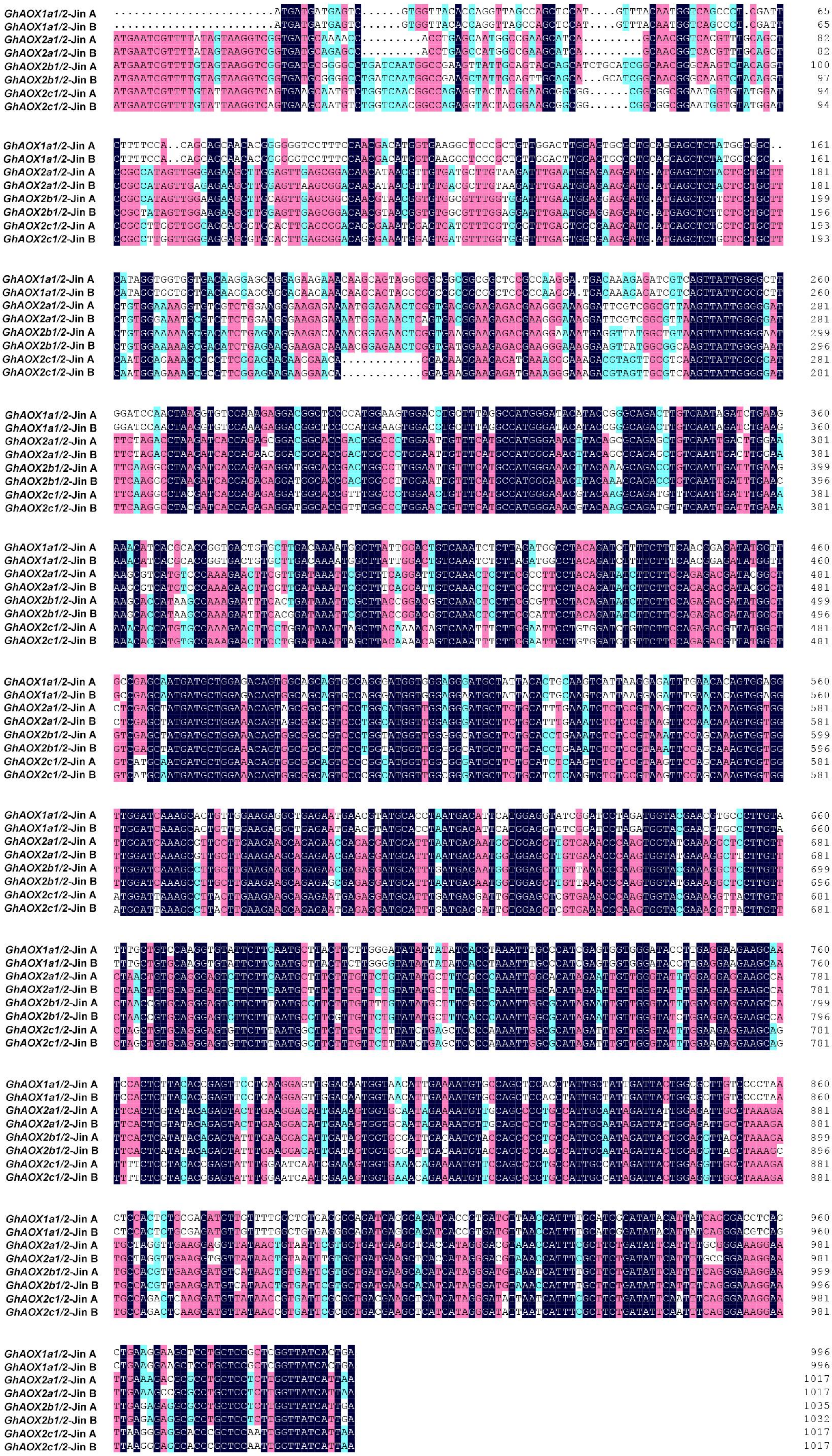

Supplemental Figure S6.

The sequence comparison of *GhAOX* family members in Jin A and Jin B. Black boxes indicated identical base sequence.

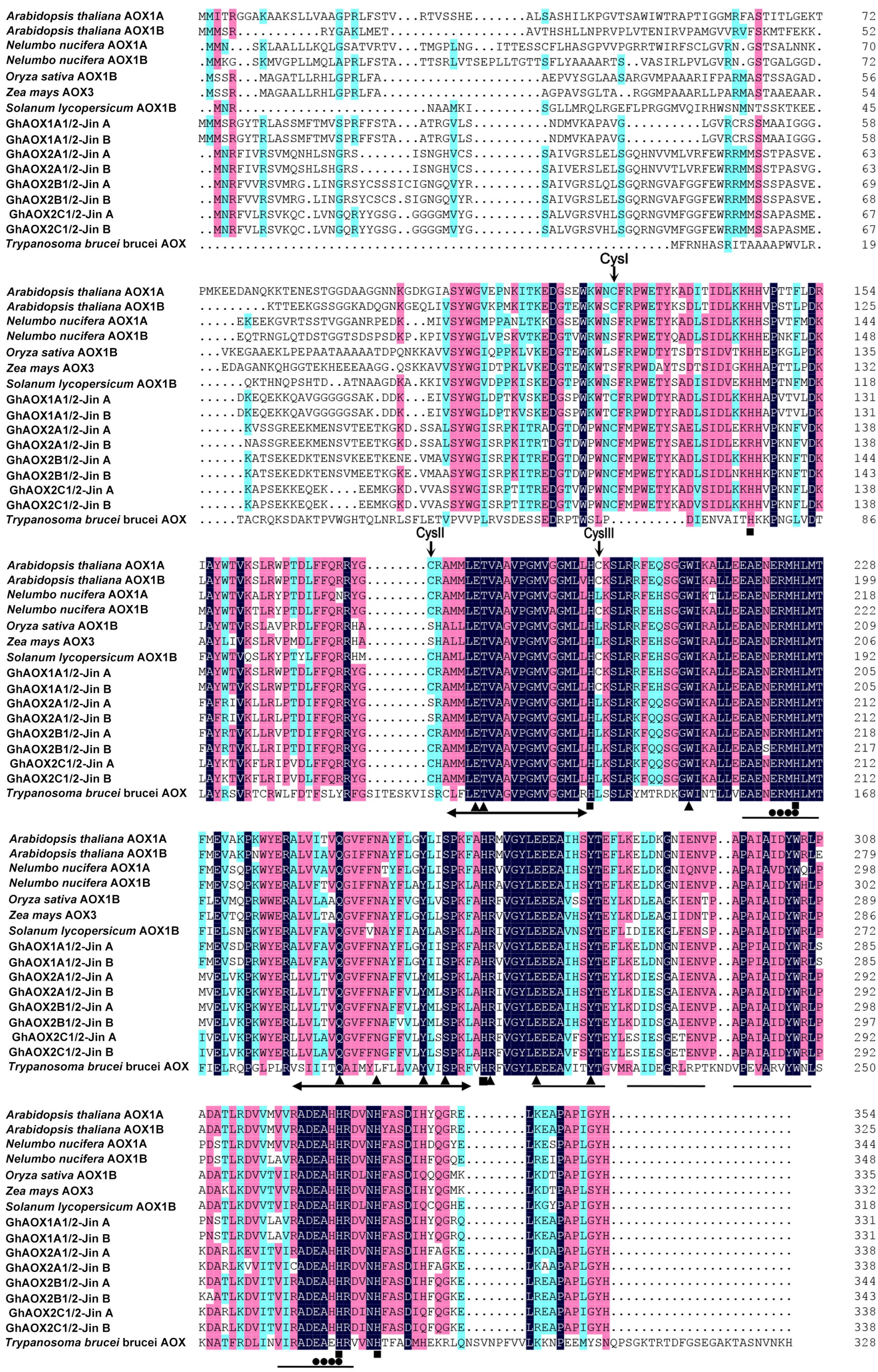

Supplemental Figure S7.

Multiple sequence alignment of GhAOX proteins with homologous sequences from other plant species. Black shading indicates strictly conserved amino acid residues, whereas pink and blue shading denote residues with similar physicochemical properties. Conserved cysteine residues

(Functionally critical conserved amino acid residues, ▲), and histidine residues (■) are specifically highlighted. Predicted helical regions potentially involved in the assembly of the hydroxo-bridged binuclear iron center are underlined. Conserved iron-binding motifs (EXXH, ●) are also annotated. Residue regions predicted to form putative transmembrane α-helices are marked with double-headed arrows.

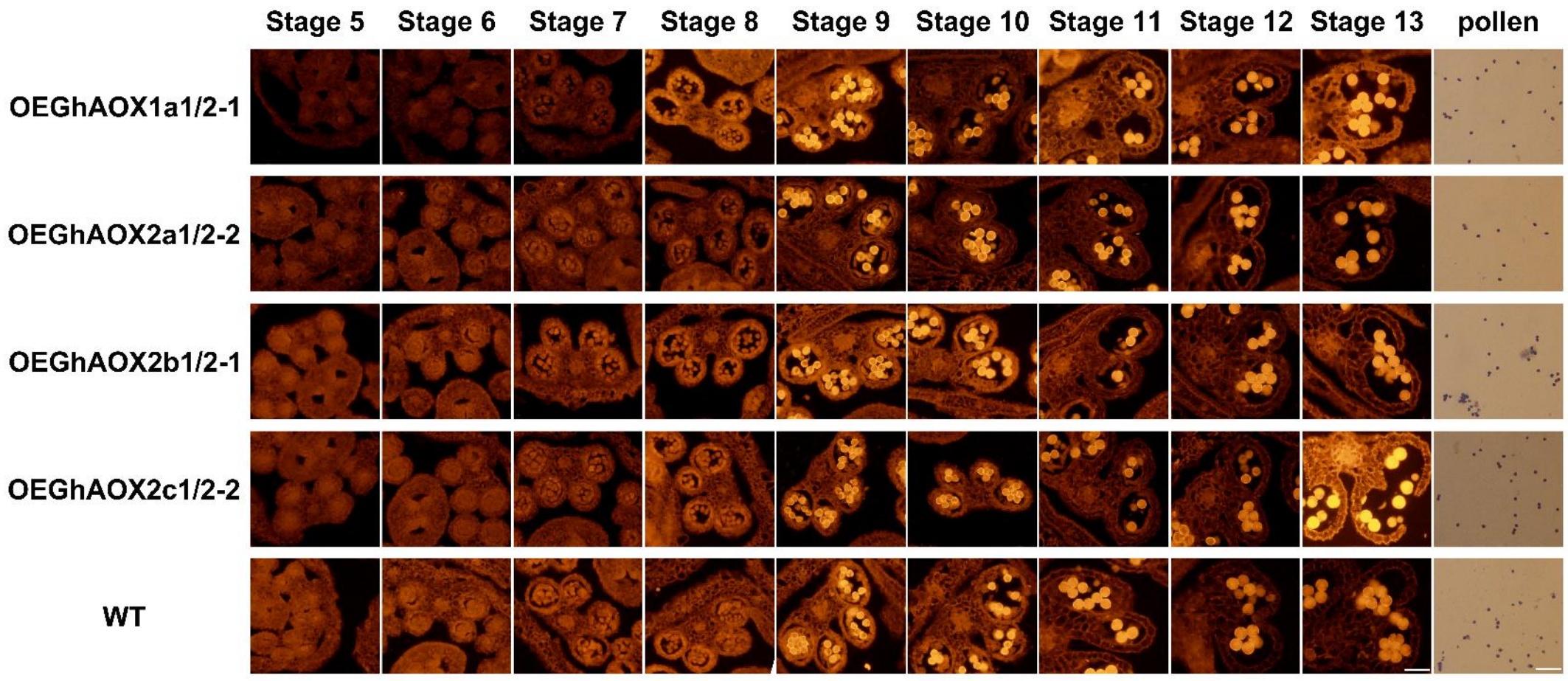

Supplemental Figure S8.

Stamen development and pollen morphology in *GhAOX*-transgenic *Arabidopsis thaliana*. Anther histological sections were prepared via conventional paraffin embedding and stained with propidium iodide (scale bar = 40 μm). Mature pollen grains were stained with I_2-KI solution for viability observation (scale bar = 80 μm). Stamen developmental stages were classified according to the criteria described by Sanders et al. (1999). No obvious morphological differences in stamen development were detected between *GhAOX*-overexpressing lines and wt plants, consistent with the comparable pollen staining intensities observed via I_2-KI staining.

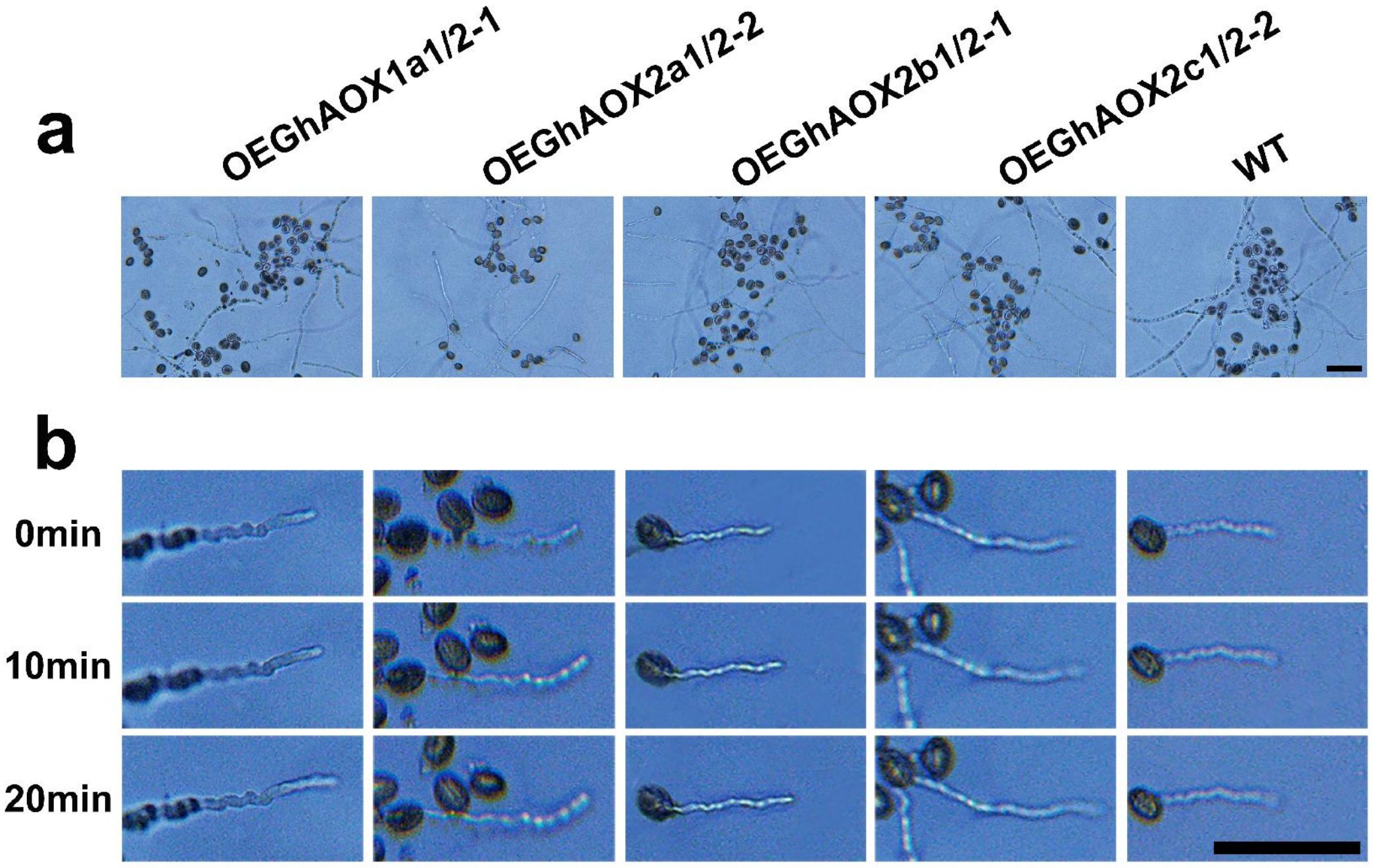

Supplemental Figure S9.

Pollen germination assay of *GhAOX*-transgenic *Arabidopsis thaliana*. **(a)** Pollen germination status of transgenic lines after 8 h of incubation on solid germination medium (scale bar = 100 μm). **(b)** Dynamic growth of pollen tubes within 20 min following the initiation of pollen germination (scale bar = 100 μm).

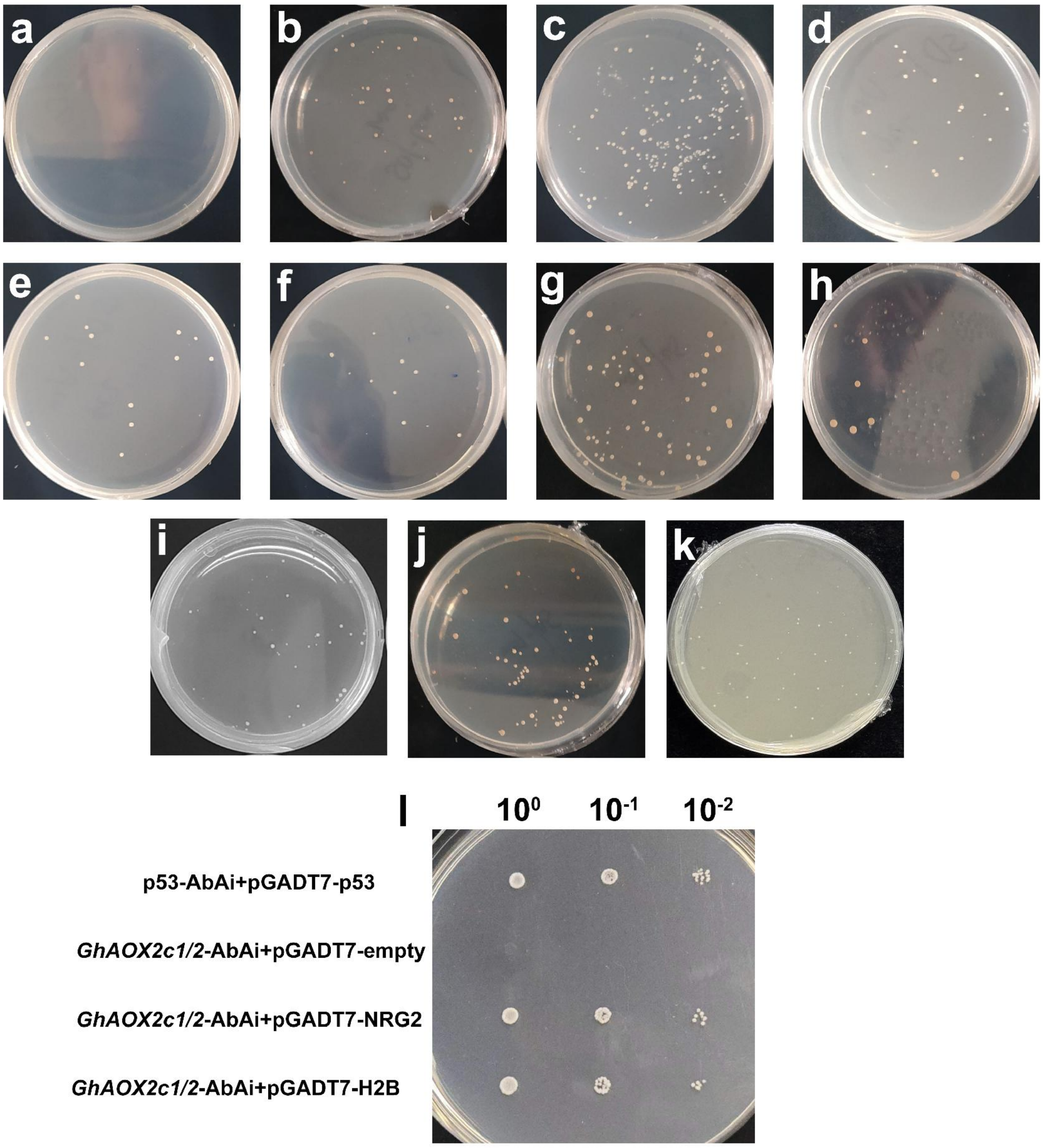

Supplemental Figure S10.

Yeast one-hybrid (Y1H) verification of protein-promoter interactions. **(a)** Y1H reporter yeast strains carrying the *GhAOX2c1/2* promoter were transformed with empty pGADT7 vector and cultured on SD/-Ura/-Leu medium supplemented with 200 ng/mL AbA. **(b-k)** The same *GhAOX2c1/2* promoter reporter strains were separately transformed with positive pGADT7 recombinant vectors harboring ten candidate interacting proteins, including Cell Division Control Protein 2 Homolog D, RGF1-Inducible Transcription Factor 1, ENHANCER OF AG-4 Protein 2, Adagio Protein 1, Nuclear Transport Factor 2B, Putative Transcription Elongation Factor SPT5 Homolog 1, Nitrate Regulatory Gene 2 Protein, uncharacterized proteins, and Histone H2B, followed by cultivation on SD/-Ura/-Leu medium containing 200 ng/mL AbA. **(l)** Further verification of the interaction between *GhAOX2c1/2* and key candidate proteins (Nitrate Regulatory Gene 2 [NRG2] and Histone H2B). The *GhAOX2c1/2*-AbAi reporter strain was transformed with pGADT7-NRG2 and pGADT7-Histone H2B, respectively, and cell growth was observed on SD/-Ura/-Leu medium supplemented with 200 ng/mL AbA. The p53-AbAi strain

transformed with pGADT7-p53 was used as the positive control, while the *GhAOX2c1/2*-AbAi strain transformed with empty pGADT7 vector served as the negative control.

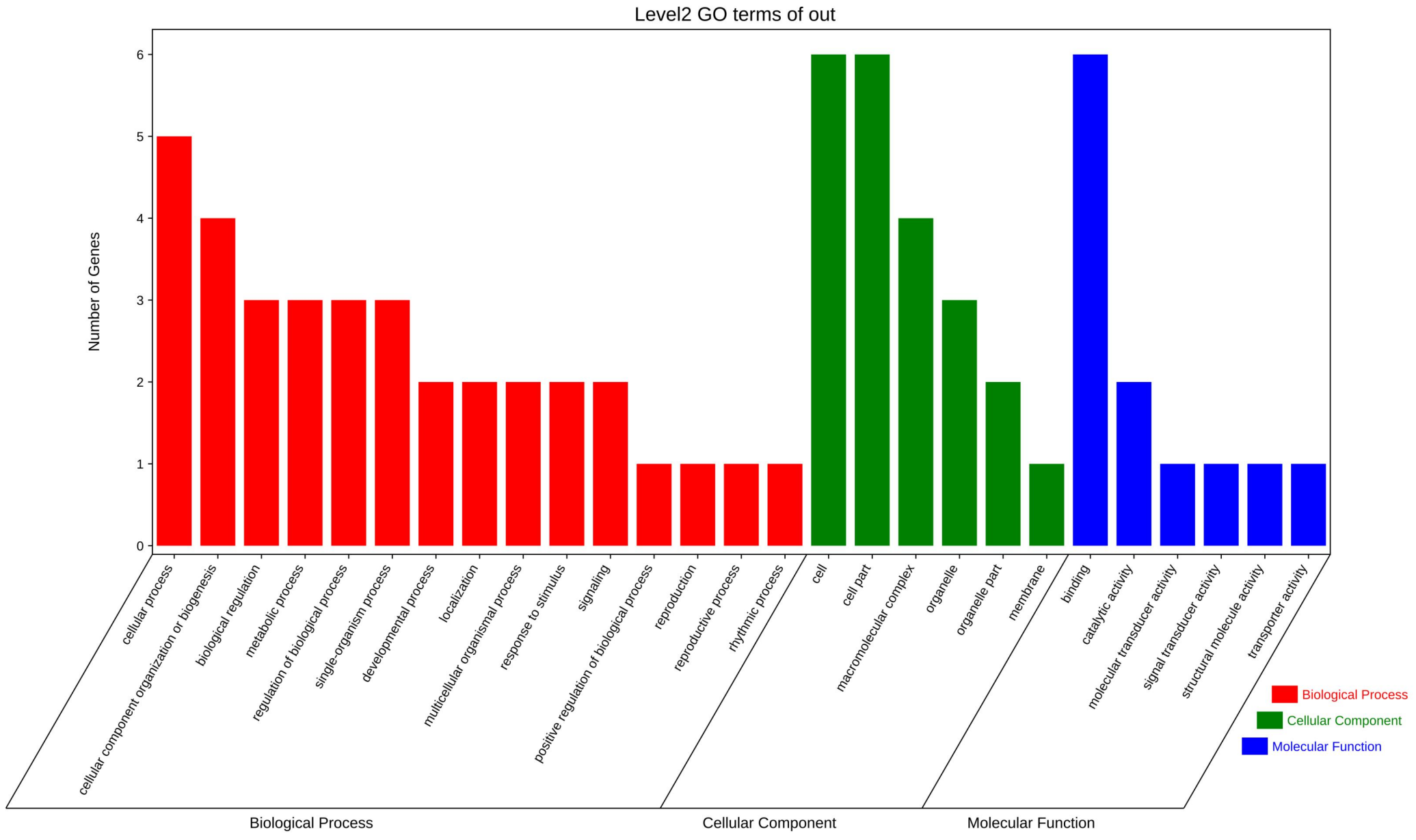

Supplemental Figure S11.

GO analysis of 10 positive sequences located in the nucleus.

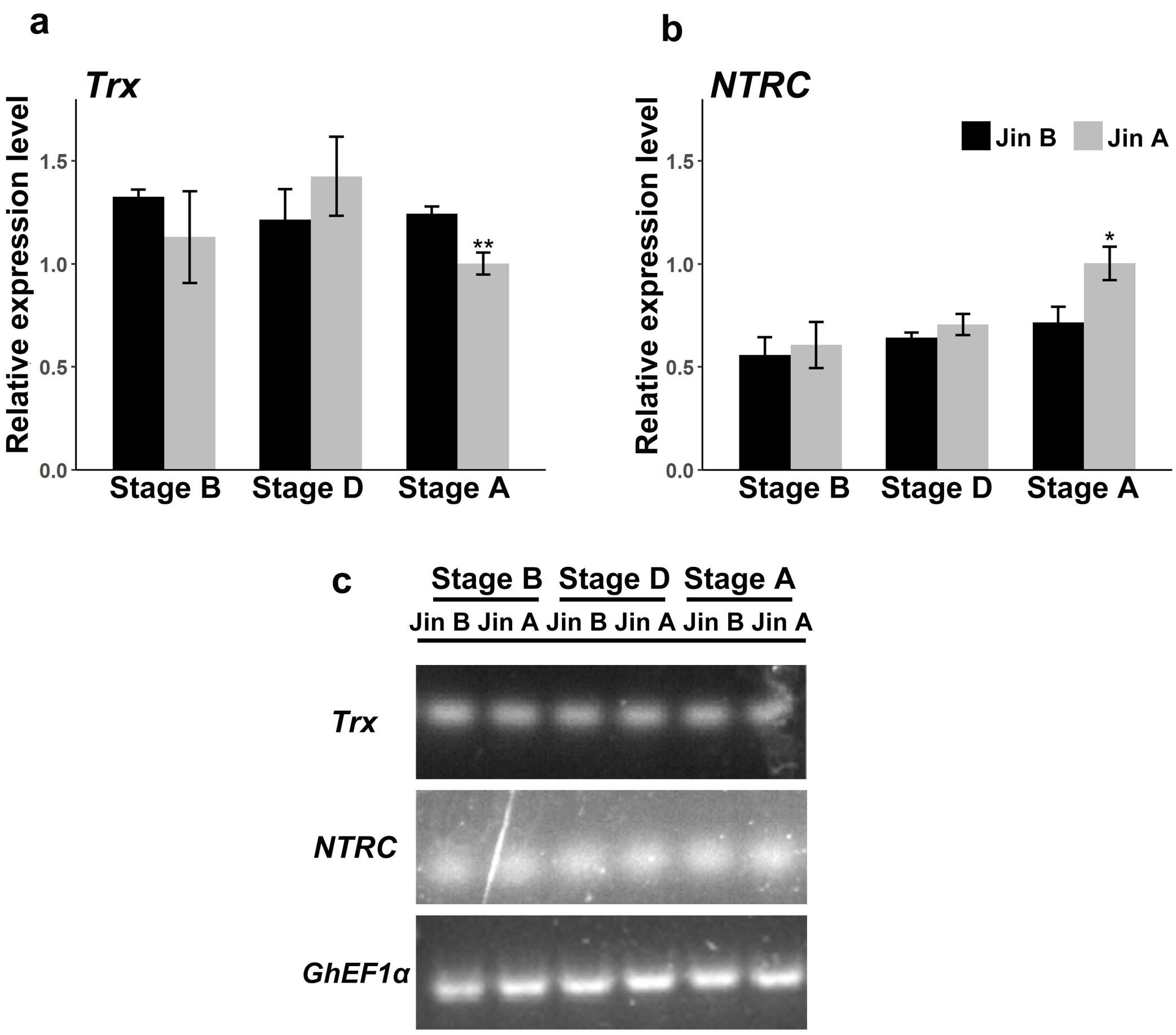

Supplemental Figure S12.

Transcript abundance of *Trx* and *NTRC* during anther development in Jin A and Jin B. qRT-PCR **(a & b)** and RT-PCR **(c)** revealed no significant differences in the expression levels of *Trx* and *NTRC* at Stage D. The cotton *GhEF1α* was employed as an internal reference for transcript normalization. All quantitative data are presented as mean ± SD from three independent experiments. Asterisks indicate statistically significant differences between Jin A and Jin B (**P* < 0.05, ***P* < 0.01; Student's *t*-test).
