# Supplementary material for: Genome-wide identification of GhAOX gene family and its expression and regulation in cotton CMS line Jin A
Source: Front Plant Sci. 2026 Aug 14;17:1898123. doi: 10.3389/fpls.2026.1898123 (PMC13523418; doi:10.3389/fpls.2026.1898123)
Supplement: Supplementary file 3 [file Table1.doc]

**Supplemental Table S1.** Primer sequence

| Primer for CDS amplification of *GhAOX* family members | |
| --- | --- |
| Name | sequence |
| GhAOX1a1/2-F | GGGATGATGATGAGTCGTGG |
| GhAOX1a-R | TTCAGTGATAACCGAGCGGA |
| GhAOX2a-F | AAAGAGAGGGAAGAAAATGAATCGT |
| GhAOX2a-R | GTTTGCATCAAGCACGAACCT |
| GhAOX2b-F | AGCGCAAAAGAAGAAGAAAATGA |
| GhAOX2b-R | ACAGATTCTACACGATCCAATATCA |
| GhAOX2c-F | AAGAGAGAAGAAAATGAATCGT |
| GhAOX2c-R | ACATAGAATTGAGTTAATGATAACC |
| Primer for qRT-PCR of *GhAOX* family members | |
| Name | sequence |
| GhEF-1α-F | agaccaccaagtactactgcac |
| GhEF-1α-R | ccaccaatcttgtacacatcc |
| GhAOX1a-F | GTGGACCTGCTTTAGGCCAT |
| GhAOX1a-R | TGCTCGGCAACCATATCTCC |
| GhAOX2a-F | ATGGTGGAGCTTGTGAAACCC |
| GhAOX2a-R | AGGGGCTGCAACATTTTCTATTG |
| GhAOX2b-F | AACGTAACGGTGTGGCGTTT |
| GhAOX2b-R | TCACCGAGTTCTCCGTTTTGT |
| GhAOX2c-F | AGAGGTACTACGGAAGCGGC |
| GhAOX2c-R | GAGCTCATCATCCTTCGCCA |
| GhAOX4-F | TGAGTCTCTCTTCATCGGCTT |
| GhAOX4-R | GAGTTGCAACGCAAAGGGTT |
| Primer for promoter of *GhAOX* family members | |
| Name | sequence |
| GhAOX1a-UP-F | CCCAAAATTTGAAGAAACGTAACCA |
| GhAOX1a-UP-R | CCTGGTGTAACCACGACTCA |
| GhAOX2a-UP-F | CGCAACGGTTCTGATGATGAC |
| GhAOX2a-UP-R | TTTGCATCACCGACCTTACTA |
| GhAOX2b-UP-F | GAAGGACATGACACCCTCGC |
| GhAOX2b-UP-R | CGCATCACCGACCTTACTACA |
| GhAOX1c-UP-F | GCAGCACCTATGATTTTTCTGACA |
| GhAOX1c-UP-R | GCTTCCGTAGTACCTCTGGC |
| pAbAi-*GhAOX* seamless cloning primer sequence | |
| Name | sequence |
| 1a-AbA-F | ATGAATTGAAAAGCTATTTGACCATCAATTTTTTAAAAAGAGTTAGAT |
| 1a-AbA-R | GAGCACATGCCTCGAGAAATGCACGTAAGCTGTTATG |
| 2a-AbA-F | ATGAATTGAAAAGCTTTGGCGGCAATTATTGTTTCTCG |
| 2a-AbA-R | GAGCACATGCCTCGAAAGCGTCAACCTATTATGACGT |
| 2b-AbA-F | ATGAATTGAAAAGCTTTCAGGGGTTAAACATGATATTAACCCA |
| 2b-AbA-R | GAGCACATGCCTCGAACGGCCACGAGTCTC |
| 2c-AbA-F | ATGAATTGAAAAGCTAATTTAACTTCAAATTGCTCTAATTTTAAATTTATCTT |
| 2c-AbA-R | GAGCACATGCCTCGACGAAAGGACCTAATGAACA |
| *TrxO1* and *NTRC* qRT-PCR [primer sequence](javascript:;) | |
| Name | Sequence |
| TrxO1-F | GTGCCGGTTCCAGAGAATGT |
| TrxO1-R | GGAGCACACCATGTAGCAGT |
| NTRC-F | GTTTCCACTCTCCTCCCACG |
| NTRC-R | AGCAGGACCTGAGCCTATGA |
| Prokaryotic expression of linking primers [sequence](javascript:;) | |
| Name | Sequence |
| GhAOX1a-pET-F | GCCCAGCCGGCGATGGCCATGATGATGAGTCGTGGTTACACCAGG |
| GhAOX1a-pET-R | TCTCAGTGGTGGTGGTGGTGGTGGTGATAACCGAGCGGAGCAGGA |
| pET-GhAOX1a-F | TCCTGCTCCGCTCGGTTATCACCACCACCACCACCACCACTGAGA |
| pET-GhAOX1a-R | CCTGGTGTAACCACGACTCATCATCATGGCCATCGCCGGCTGGGC |
| GhAOX2a/b-pET-F | GTGCGGCCGCAAGCTTATGATAACCAAGAGGAGCAGGCG |
| GhAOX2a-pET-R | GAATTAATTCGGATCCTATGAATCGTTTTATAGTAAGGTCGGTGATGCA |
| GhAOX2b-pET-R | GAATTAATTCGGATCCTATGAATCGTTTTGTAGTAAGGTCGGTGA |
| GhAOX2c-pET-F | GTGCGGCCGCAAGCTTATGATAACCAATTGGAGCGGGTG |
| GhAOX2c-pET-R | GAATTAATTCGGATCCTATGAATCGTTTTGTATTAAGGTCAGTGAAGCAAT |
| pRI101-AN-*GhAOXs* Seamless cloning [primer sequence](javascript:;) | |
| Name | Sequence |
| GhAOX1a-RI101-F | TTCAGAATTCGGATCTCAGTGATAACCGAGCGGAGCA |
| GhAOX1a-RI101-R | CACTGTTGATACATAATGATGATGAGTCGTGGTTACACCAG |
| GhAOX2a-RI101-F | TTCAGAATTCGGATCTTAATGATAACCAAGAGGAGCAGGCG |
| GhAOX2a-RI101-R | CACTGTTGATACATAATGAATCGTTTTATAGTAAGGTCGGTGATG |
| GhAOX2b-RI101-F | TTCAGAATTCGGATCTCAATGATAACCAAGAGGAGCAGGC |
| GhAOX2b-RI101-R | CACTGTTGATACATAATGAATCGTTTTGTAGTAAGGTCGGTGA |
| GhAOX2c-RI101-F | TTCAGAATTCGGATCTTAATGATAACCAATTGGAGCGGGTGC |
| GhAOX2c-RI101-R | CACTGTTGATACATAATGAATCGTTTTGTATTAAGGTCAGTGAAGC |
| pTRV2-*GhAOXs* seamless cloning [primer sequence](javascript:;) | |
| Name | Sequence |
| GhAOX1a-pTRV2-F | TAAGGTTACCGAATTCATGATGATGAGTCGTGGTTACACCAG |
| GhAOX1a-pTRV2-R | GCTCGGTACCGGATCCCTAAAGCAGGTCCACTTCCAT |
| GhAOX2a-pTRV2-F | TAAGGTTACCGAATTCTGCAAAACCACCTGAGCAATGGC |
| GhAOX2a-pTRV2-R | GCTCGGTACCGGATCCAATCCTGAAAGCGAATTTATCAACGAAGT |
| GhAOX2b-pTRV2-F | TAAGGTTACCGAATTCTGCGGGGCCTGATCAATGG |
| GhAOX2b-pTRV2-R | GCTCGGTACCGGATCCCTTATGGTGCTTCTTCAAATCAATTGACAGG |
| GhAOX2c-pTRV2-F | TAAGGTTACCGAATTCAGCAATGTCTGGTCAACGGC |
| GhAOX2c-pTRV2-R | GCTCGGTACCGGATCCTGTTTTGTAAGCTAATTTATCCAGGAAGTTCT |
| *Arabidopsis* internal reference primers | |
| Name | Sequence |
| AtACTIN8-qF | ATGACTCAGATCATGTTTGAGACC |
| AtACTIN8-qR | TCAGTAAGGTCACGACCAGCAA |

**Supplemental Table S2.** *AOX* prokaryotic expression induction conditions

|  | Induction temperature (℃) | Induction time (h) | IPTG final concentrations (mmol/L) | OD_600_ value |
| --- | --- | --- | --- | --- |
| GhAOX1A1/2 | 25 | 5 | 0.2 | 0.8 |
| GhAOX2A1/2 | 25 | 5 | 0.2 | 0.8 |
| GhAOX2B1/2 | 24 | 6 | 0.4 | 0.8 |
| GhAOX2C1/2 | 25 | 5 | 0.2 | 0.8 |

**Supplemental Table S3.** Ka, Ks, Ka/Ks distribution of *GhAOX* gene pairs

| Seq_1 | Seq_2 | Ka | Ks | Ka**/**Ks |
| --- | --- | --- | --- | --- |
| *GhAOX2b1* | *GhAOX2c1* | 0.1076 | 0.7092 | 0.1518 |
| *GhAOX2a1* | *GhAOX2a2* | 0.0434 | 0.0918 | 0.4733 |
| *GhAOX2a1* | *GhAOX2c2* | 0.1388 | 0.6730 | 0.2062 |
| *GhAOX2c1* | *GhAOX2a2* | 0.1334 | 0.7465 | 0.1787 |
| *GhAOX2c1* | *GhAOX2c2* | 0.0116 | 0.0641 | 0.1812 |
| *GhAOX4a1* | *GhAOX4a2* | 0.0156 | 0.0524 | 0.2988 |
| *GhAOX1a1* | *GhAOX1a2* | 0.0078 | 0.0369 | 0.2106 |
| *GhAOX2a2* | *GhAOX2c2* | 0.1402 | 0.6807 | 0.2060 |

Supplemental Table S4. ROS levels of transgenic A. thaliana leaves

|  | Superoxide anion content (μg/Fwg) | Hydrogen peroxide content (μmol/Fwg) |
| --- | --- | --- |
| OEGhAOX1a1/2-1 | 53.9±5.0 | 1.17±0.10* |
| OEGhAOX2a1/2-2 | 43.7±10.2 | 1.42±0.20 |
| OEGhAOX2b1/2-1 | 50.7±1.3 | 1.39±0.20 |
| OEGhAOX2c1/2-2 | 43.8±5.5* | 1.14±0.02** |
| wt | 57.2±4.1 | 1.44±0.08 |

All data are presented as the means ± SD of three independent biological replicates. Asterisks represent statistically significant differences between transgenic *Arabidopsis* and wt control (* P < 0.05, ** P < 0.01; Student’s t-test).

**Supplemental Table S5.** Germination rate, pollen tube growth rate, and pollen tube width of transgenic *Arabidopsis* pollen.

|  | Pollen germination rate (%) | Pollen tube growth rate (μm/min) | Pollen tube width (μm) |
| --- | --- | --- | --- |
| OEGhAOX1a1/2-1 | 70.0±0.4** | 13.9±3.1* | 7.2±1.3 |
| OEGhAOX2a1/2-2 | 70.9±2.2* | 9.5±2.6 | 8.0±0.5 |
| OEGhAOX2b1/2-1 | 78.6±3.6 | 11.0±2.6 | 6.6±1.0 |
| OEGhAOX2c1/2-2 | 76.0±5.1 | 14.2±4.5 | 7.2±0.5 |
| wt | 78.4±2.2 | 7.5±0.9 | 8.1±0.5 |

All data are presented as the means ± SD of three independent biological replicates. Asterisks represent statistically significant differences between transgenic *Arabidopsis* and wt control (* P < 0.05, ** P < 0.01; Student’s t-test).

**Supplemental Table S6.** Functions and locations of 10 positive sequences

| No. | ID in cotton library | ID in NCBI | Annotation | Function |
| --- | --- | --- | --- | --- |
| 1 | Ghir_D01G009690.1 | XM_016850906.2 | Cell division control protein 2 homolog D | Has kinase activity, plays a key role in the control of the eukaryotic cell cycle |
| 2 | Ghir_A11G008590.1 | XM_016839879.2 | RGF1 INDUCIBLE TRANSCRIPTION FACTOR 1 | plays a central role in mediating RGF1 hormone peptide signaling |
| 3 | Ghir_A10G012790.1 | XM_041102302.1 | ENHANCER OF AG-4 protein 2 | functions as a repressor of flowering, the specification of stamen and carpel identities, controls anthocyanin accumulation |
| 4 | Ghir_A01G002680.1 | XM_016868800.2 | zinc finger CCCH domain-containing protein 34 | zinc finger protein, has DNA-bingding and metal-binding function |
| 5 | Ghir_A05G000450.1 | XM_016834697.2 | adagio protein 1 | Component of an E3 ubiquitin ligase complex that plays a central role in blue light-dependent circadian cycles. |
| 6 | Ghir_A05G032090.1 | XM_041112591.1 | nuclear transport factor 2B | Facilitates protein transport into the nucleus. Interacts with various nucleoporins and with Ran-GDP. |
| 7 | Ghir_A05G027490.1 | XM_041112196.1 | putative transcription elongation factor SPT5 homolog 1 | May regulate transcription elongation by RNA polymerase II. |
| 8 | Ghir_A05G024110.1 | XM_016854631.2 | Nitrate regulatory gene2 protein | Required for nitrate signaling. Regulates expression of the nitrate-responsive genes NIA1, NIR1, NRT2.1 and NPF6.3/NRT1.1. |
| 9 | Ghir_D09G024840.1 | XM_041101456.1 | uncharacterized | zinc finger protein, has metal-binding function |
| 10 | Ghir_A08G000370.1 | XM_016900499.2 | histone H2B | Histones thereby play a central role in transcription regulation, DNA repair, DNA replication and chromosomal stability. |

**Supplemental Table S7.** Expression analysis of 10 positive sequences

| Gene_ID | B2 | K2 | B3 | K3 |
| --- | --- | --- | --- | --- |
| Cell division control protein 2 homolog D | 107.82 | 84.63 | 84.71 | 84.50 |
| RGF1 INDUCIBLE TRANSCRIPTION FACTOR 1 | 5.52 | 4.17 | 3.91 | 3.15 |
| ENHANCER OF AG-4 protein 2 | 16.48 | 19.68 | 17.96 | 12.94 |
| zinc finger CCCH domain-containing protein 34 | 27.76 | 29.48 | 26.60 | 22.69 |
| adagio protein 1 | 23.24 | 21.82 | 24.15 | 19.54 |
| nuclear transport factor 2B | 17.84 | 23.81 | 16.98 | 23.17 |
| putative transcription elongation factor SPT5 homolog 1 | 10.60 | 12.06 | 10.55 | 11.43 |
| Nitrate regulatory gene2 protein | 23.12 | 12.62 | 23.24 | 11.30 |
| uncharacterized | 7.28 | 10.04 | 8.91 | 9.35 |
| histone H2B | 435.81 | 542.45 | 368.30 | 526.21 |

Expression levels were derived from previously published transcriptome data (Yang et al., 2014) and presented as log2(FPKM+1) values. B2, Sporogenous cells stage of Jin A; B3, Microsporocyte stage of Jin A; K2, Sporogenous cells stage of Jin B; K3, Microsporocyte stage of Jin B.
